# ShinyVar: a web-based application for comparative Influenza variant analysis supporting structure-guided approaches to vaccine and antiviral drug design

**DOI:** 10.7717/peerj.21158

**Published:** 2026-06-08

**Authors:** Danusorn Lee, Unitsa Sangket

**Affiliations:** 1 Division of Biological Science, Faculty of Science, Prince of Songkla University, Hat Yai, Songkhla, Thailand; 2 Center for Genomics and Bioinformatics Research, Faculty of Science, Prince of Songkla University, Hat Yai, Songkhla, Thailand

**Keywords:** Influenza viruses, Influenza vaccines, Influenza variants, Shiny app, Drug design, Protein modeling, Molecular docking

## Abstract

**Background:**

Influenza viruses remain a significant global health threat due to their high mutation rates and extensive subtype diversity. Genetic variability promotes the mixing of viral subpopulations and facilitates the emergence of novel, potentially virulent, strains. Seasonal influenza vaccines, typically targeting the A/H1N1pdm09 subtype, A/H3N2 subtype, B/Victoria lineage, and B/Yamagata lineage must be reformulated annually, yet their effectiveness and antiviral resistance are continually challenged by antigenic drift. Notably, the H275Y mutation confers a marked reduction in sensitivity to oseltamivir. Comparative variant analysis provides an effective approach for understanding these genetic variations, which is essential for enhancing vaccine effectiveness and predicting drug resistance. Existing tools enable pairwise comparison of variant call format (VCF) files through command-line interfaces but a major limitation is that they only merge variants, and users must manually group variants across multiple samples.

**Methods:**

Here, we present ShinyVar, a web-based application developed using the R Shiny framework for comparative variant analysis. The application automatically identifies and visualizes mutations in influenza viruses (IFVs) through interactive comparison of variant profiles across subpopulations. The analysis included the A/Wisconsin/67/2022 (H1N1)pdm09-like virus, A/District of Columbia/27/2023 (H3N2)-like virus, B/Austria/1359417/2021 (B/Victoria lineage)-like virus, and B/Phuket/3073/2013 (B/Yamagata lineage)-like virus as representative subtypes for comparison across the past four years.

**Results:**

Variant analysis revealed 181 unique variants in B/Victoria lineage 2025, 101 unique variants in A/H1N1pdm09 2024, 37 unique variants in A/H3N2 subtype 2024, and seven unique variants in B/Yamagata lineage 2025. Structural analysis focused on HA_A/H1N pdm091, NA_A/H1N1 pdm09, and NA_B/Victoria. In HA_A/H1N1pdm09, eight non-synonymous mutations were identified. For downstream structural analysis, p.Thr137Ala or p.Thr120Ala mature numbering led to the loss of a hydrogen bond within the 5J8 antibody binding site, resulting in a slight decrease in binding affinity. In the NA_B/Victoria lineage, eleven non-synonymous mutations marginally disrupted key oseltamivir interaction residues (E276 and R292), resulting in lower binding affinity compared with NA_A/H1N1pdm09.

**Conclusions:**

ShinyVar is a fast and efficient tool for variant detection and visualization, making it suitable for monitoring not only IFV evolution but also the evolution of other pathogens. ShinyVar facilitates the identification of novel, shared, and unique variants linked to important traits, including drug resistance and virulence under selective pressure, contributing to improved insights into pathogen evolution and the rational design of vaccines and antiviral drugs.

## Introduction

The influenza virus (IFV), a rapidly evolving member of the Orthomyxoviridae family, is responsible for seasonal epidemics and occasional pandemics. Its high mutation rate leads to a mixing of viral subpopulations, which favors the emergence of novel and potentially virulent subtypes. Genetic divergence within IFV populations is determined by host immune pressure and cellular factors (Beigi, 2014). Of the four influenza types, types A and B remain the most likely to become pandemic, and continue to evolve through genetic drift (Taubenberger & Morens, 2010; Zhou et al., 2023). For the Southern Hemisphere year 2025, the recombinant-based vaccines for the A/Wisconsin/67/2022 (H1N1)pdm09-like virus, A/District of Columbia/27/2023 (H3N2)-like virus, B/Austria/1359417/2021 (B/Victoria lineage)-like virus, and B/Phuket/3073/2013 (B/Yamagata lineage)-like virus were updated before the influenza season (World Health Organization, 2024b). For the Northern Hemisphere year 2024–2025, the recombinant-based vaccines for the A/Wisconsin/67/2022 (H1N1)pdm09-like virus, A/Massachusetts/18/2022 (H3N2)-like virus, B/Austria/1359417/2021 (B/Victoria lineage)-like virus, and B/Phuket/3073/2013 (B/Yamagata lineage)-like virus were also updated before the influenza season (World Health Organization, 2024a). However, this process faces the persistent challenge of antigenic variation. IFVs gradually accumulate point mutations during annual circulation, largely due to the high error rate of viral RNA-dependent RNA polymerase (Lin et al., 2019). As with the pandemic H1N1pdm09 subtype, genetic analyses revealed that the 2009 H1N1 influenza virus originated from animal influenza strains and was distinct from the human seasonal H1N1 viruses circulating since 1977. Following its emergence in North America in April 2009, the virus spread rapidly worldwide, prompting the WHO to declare a pandemic by June after confirmed cases were reported in 74 countries. Today, the H1N1 (2009) strain continues to circulate as a seasonal influenza virus and is included in annual influenza vaccines (World Health Organization, 2010).

The subtyping of type A IFVs is based on two surface proteins: hemagglutinin (HA) and neuraminidase (NA). For example, H1N1 refers to H1 hemagglutinin and N1 neuraminidase. In contrast, type B IFVs are not divided into subtypes but are instead classified into two lineages: B/Yamagata and B/Victoria, because influenza B viruses generally change more slowly in their genetic and antigenic properties than influenza A viruses. Moreover, B/Yamagata viruses have not circulated globally since March 2020 (U.S. Centers for Disease Control and Prevention, 2025).

The accumulation of mutations forces the constant updating of vaccines and surveillance of the evolution of antiviral resistance. An example of antiviral resistance in the H1N1pdm09 subtype was investigated in a recent study of the widely used antiviral drug oseltamivir, which inhibits the active site of NA, an enzyme that is crucial for the release of the virus from infected cells (Davies, 2010). The resistance was found to be conferred by the H275Y mutation in the NA gene (Behillil et al., 2020). An earlier study of the H1N1pdm09 subtype showed that the human 5J8 antibody targeted the receptor binding site (RBS) at the head of the HA protein (Hong et al., 2013). The 5J8 antibody has since become a valuable tool in neutralization studies and vaccine development.

The continuous surveillance of circulating IFV subtypes and evaluation of viral evolution to detect the emergence of virulent or drug-resistant variants are critical for seasonal vaccine composition and public health preparedness (Sangket et al., 2015). The key to understanding viral subpopulations associated with pathogenic traits is the efficient identification of signature mutations or single nucleotide polymorphisms (SNPs). This task is made easier by the extensive sequence data made available by next-generation sequencing (NGS). NGS is a high-throughput sequencing technique capable of generating large genomic datasets, which are used to investigate genetic diversity in viral populations (Grada & Weinbrecht, 2013). The alignment of sequence reads from NGS to reference genomes can be processed through variant calling pipelines such as Genome Analysis Toolkit (GATK) (McKenna et al., 2010) and BWA Aligner (Md et al., 2019), producing variant call format (VCF) files that summarize variants, including SNPs and small indels (Lee & Sangket, 2024). VCF is considered the gold standard for variant analysis because it stores detailed information about genetic variations such as SNPs, insertions, deletions, and structural variants relative to a reference genome. The VCF is a tab-delimited, text-based file format capable of representing multiple variants across large genomic regions. Importantly, the INFO column in a VCF file can include annotated information generated by annotation software such as SnpEff (Cingolani et al., 2012).

Comparative variant analysis using VCF files can be used to compare genetic variants across different samples, across results generated by different tools, or in a reference genome. It is commonly used to validate variant calling results, evaluate the performance of new analytical pipelines, and investigate genetic differences within populations or disease contexts (de Schaetzen Van Brienen et al., 2020). Additionally, combining or comparing variants detected by multiple callers can improve the accuracy of variant identification (Liang et al., 2019). Existing tools such as BEDTools (Quinlan & Hall, 2010) and VCFtools (Danecek et al., 2011) enable pairwise comparison of VCF files through command-line interfaces. Although VCFtools is available through a graphical user interface (Galaxy) (The Galaxy Community, 2024), the size of samples that can be processed is often limited by the space available on the Galaxy server. A major limitation is that these tools only merge variants, and users must manually group variants across multiple samples (*e.g*., reporting only variants present in the first sample). Consequently, these tools do not offer automatic interactive visualization for comparative variant analysis.

In this study, we present the development of ShinyVar, an interactive, web-based application that helps in vaccine and antiviral drug design. The application automatically identifies and visualizes mutations in IFVs by comparing variant profiles across subpopulations without requiring local computational infrastructure or programming expertise. Developed using the R Shiny framework, the name “ShinyVar” reflects the purpose of the application: to provide a shiny, intuitive interface for comparing and exploring IFV variants.

## Materials AND methods

### Dataset preparation

Four IFV subtypes were separated from vaccines based on cell cultures, recombinant protein or nucleic acid, as recommended by the World Health Organization (WHO). The subtypes were the A/Wisconsin/67/2022 (H1N1)pdm09-like virus, the A/District of Columbia/27/2023 (H3N2)-like virus, the B/Austria/1359417/2021 (B/Victoria lineage)-like virus and the B/Phuket/3073/2013 (B/Yamagata lineage)-like virus (World Health Organization, 2024b). The sequence read archive (SRA) samples were selected from the SRA run collector that searched for “influenza virus A” and “influenza virus B” in the NCBI SRA database (Sayers et al., 2024). However, if the SRA sample did not match the subtype, the sample was further processed with Nextclade (Aksamentov et al., 2021) to identify the source subtype. For each IFV subtype, four SRA samples were selected based on paired-end sequencing, the most recent collection dates, and stratified by subtype with one sample representing each of the past four years. Adapter sequences were trimmed with Trimmomatic v.0.39 (Bolger, Lohse & Usadel, 2014), using the parameter -phred33 ILLUMINACLIP:NexteraPE-PE.fa:2:30:10 LEADING:10 TRAILING:10 SLIDINGWINDOW:4:20 MINLEN:40 (Van Poelvoorde et al., 2022). The quality of the sequence data was checked with FastQC v. 0.12.1 (Andrews, 2010). Using bwa-mem2 (Md et al., 2019), each SRA A/H1N1pdm09 subtype paired-end read was aligned to the influenza A virus (A/Wisconsin/588/2019) (GCA_039195795.1) as the reference genome. Each SRA A/H3N2 subtype was aligned to the influenza A virus (A/Wisconsin/67/2005(H3N2)) (CY163680.1–CY163687.1). Each B/Victoria lineage subtype was aligned to the influenza B virus (B/Brisbane/60/2008) (CY115151.1–CY115158.1), and each B/Yamagata lineage subtype was aligned to the influenza B virus (B/Wisconsin/01/2010) (GCA_037902395.1).

In the next step, variant calling with GATK, filtering VCF files at a DP >= 200 (McKenna et al., 2010).

### ShinyVar pipeline

**Figure 1:**
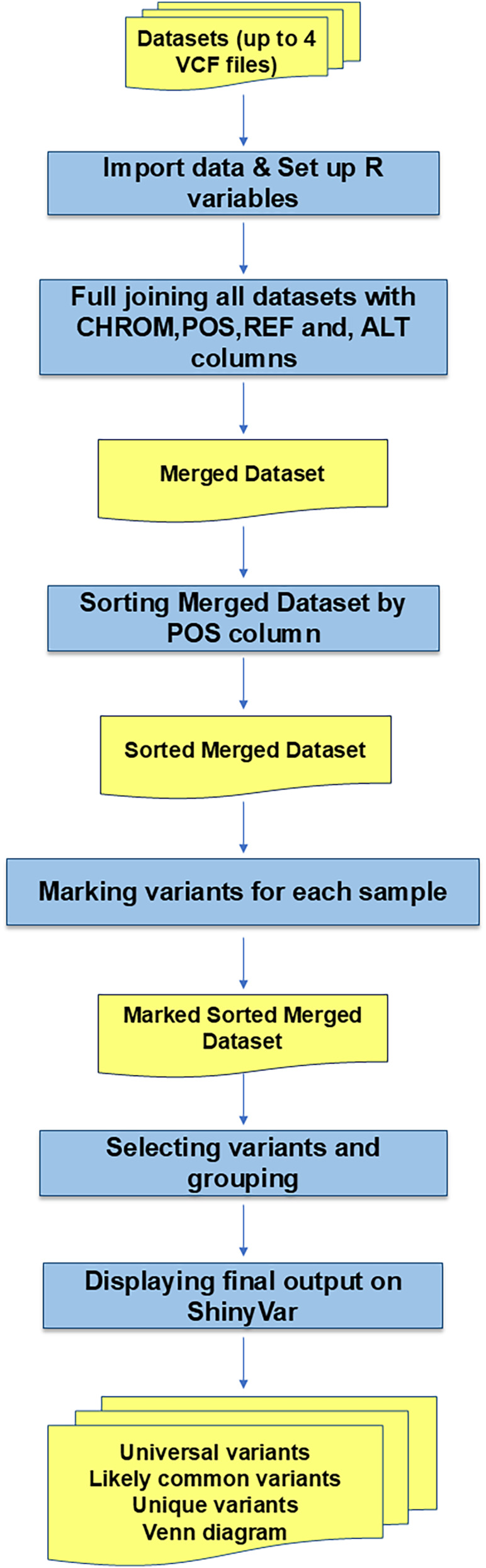
ShinyVar pipeline flowchart.

The ShinyVar pipeline (Fig. 1) serves as the core analytical engine within the ShinyVar web application. This pipeline first merged all input VCF datasets listed in Table 1 into a unified, summarized dataset using the shared variant identifiers CHROM, POS, REF, and ALT.

**Table 1:** SRA datasets information.

Subtype and lineage	Years	SRA accession number	Nextstrain Clade Clade assignment gene Subtype
A/H1N1pdm09 subtype	2024	SRR30635452	6B.1A.5a.2a HA Influenza A H1N1pdm
	2023	SRR26854354	6B.1A.5a.2 HA Influenza A H1N1pdm
	2022	SRR21931671	6B.1A.5a.1 HA Influenza A H1N1pdm
	2021	SRR14341061	6B.1A.5a.2 HA Influenza A H1N1pdm
A/H3N2 subtype	2024	SRR30252073	A NA Influenza A H3N2
	2023	SRR26854359	B.4 NA Influenza A H3N2
	2022	SRR21931765	A NA Influenza A H3N2
	2021	DRR517350	A NA Influenza A H3N2
B/Victoria lineage	2025	SRR32387634	V1A.3a.2 HA Influenza B Victoria
	2024	SRR29375162	V1A HA Influenza B Victoria
	2023	SRR27384472	V1A HA Influenza B Victoria
	2022	SRR22846271	V1A HA Influenza B Victoria
B/Yamagata lineage	2025	SRR32535134	Y1 HA Influenza B Yamagata
	2024	SRR29379495	Y1 HA Influenza B Yamagata
	2023	SRR29235600	Y1 HA Influenza B Yamagata
	2022	SRR29235613	Y1 HA Influenza B Yamagata

In brief, CHROM is the chromosome identifier, representing the chromosome or genomic segment; POS refers to the nucleotide position relative to the reference genome; REF denotes the reference nucleotide at that position; and ALT indicates the alternative nucleotide that differs from the reference. As an example, for the A/H1N1pdm09 subtype, representative VCF datasets included SRR30635452, SRR26854354, SRR21931671, and SRR14341061. The structure and format of the SRR30635452 VCF file are detailed in Table S1, providing an illustrative example of the input data used in this analysis. In the second step, the merged dataset was sorted by the POS (position) column to generate a sorted merged dataset (Table S2). In the third step, each variant entry in the sorted merged dataset was internally mapped back to the original VCF files using the CHROM, POS, REF, and ALT variant identifiers. This step established the presence of a particular variant in each sample. To achieve this, sample-specific columns (c_SRR30635452, c_SRR26854354, c_SRR21931671 and c_SRR14341061) were added to the dataset, where each cell was assigned a binary value: 1 if the variant was present in the respective sample and 0 if absent. This binary matrix representation is shown in Table 2. Finally, the variants were categorized into three distinct groups based on their distribution across samples: (1) universal variants (shared by all samples), (2) likely common variants (shared by some but not all samples), and (3) unique variants (found in only one sample). Within the ShinyVar interface, these classifications were visualized with a Venn diagram. For example, the A/H1N1pdm09 datasets revealed 1 universal variant (POS = 30), three likely common variants (POS = 80, 800, 1,287), and 1 unique variant (POS = 1,510).

**Table 2:** Marked sorted merged dataset.

CHROM	POS	REF	ALT	ID, QUAL, FILTER, INFO, FORMAT	SRR30635452	ID, QUAL, FILTER, INFO, FORMAT	SRR26854354	ID, QUAL, FILTER, INFO, FORMAT	SRR21931671	ID, QUAL, FILTER, INFO, FORMAT	SRR14341061	c_SRR30635452	c_SRR26854354	c_SRR21931671	c_SRR14341061
CY121797.1	30	TACG	T	.,1,309.01 , PASS,…	1:2,450:452:99: 15,854.0							1	0	0	0
CY121797.1	80	A	G	.,2309.01 , PASS,…	1:2,800:452:99: 15,854.0	.,3,309.01 , PASS ,…	1:2,250:452:99: 15,854.0					1	1	0	0
CY121798.1	800	T	G			.,4,309.01 , PASS ,…	1:2,250:452:99: 15,854.0	.,2,312.01 , PASS ,…	1:2,250:452:99: 15,854.0			0	1	1	0
CY121799.1	1287	T	G			.,1,309.01 , PASS ,…	1:2,250:452:99: 15,854.0	.,3,312.01 , PASS ,…	1:2,250:452:99: 15,854.0	.,4,312.01 , PASS ,…	1:2,250:452:99: 30,854.0	0	1	1	1
CY121799.1	1510	A	G	.,2309.01 , PASS ,…	1:2,250:452:99: 20,854.0	.,1,309.01 , PASS ,…	1:2,250:452:99: 15,854.0	.,3,312.01 , PASS ,…	1:2,250:452:99: 15,854.0	.,4,312.01 , PASS ,…	1:2,250:452:99: 30,854.0	1	1	1	1

**Note:**

CHROM, the chromosome identifier, representing the chromosome or genomic segment; POS, the nucleotide position relative to the reference genome; REF, the reference nucleotide at that position; ALT, indicates the alternative nucleotide that differs from the reference.

### ShinyVar interface

**Figure 2:**
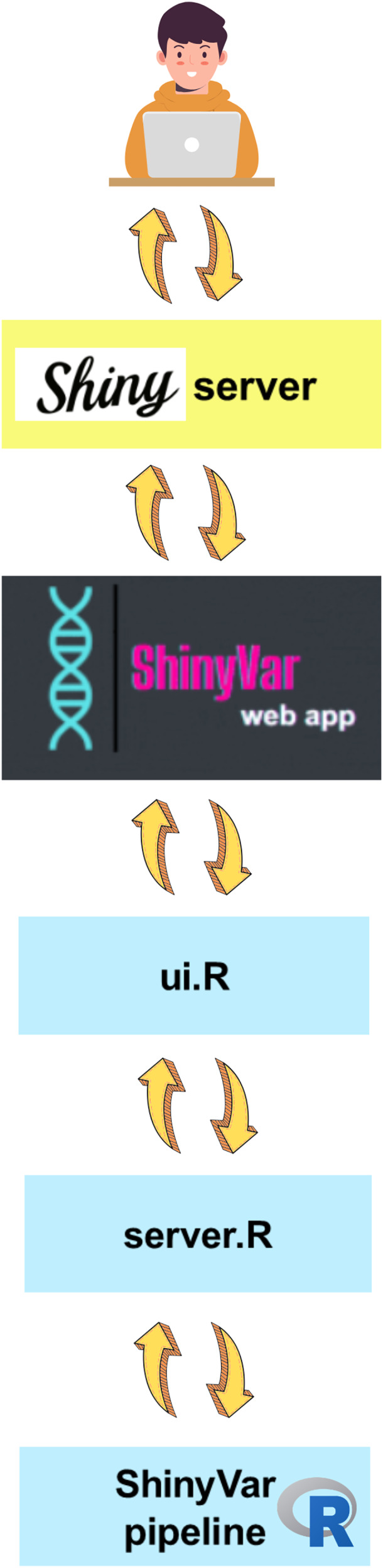
ShinyVar interface workflow. Image Credit: Canva Pro https://www.canva.com/policies/content-license-agreement/.

The ShinyVar interface workflow is shown in Fig. 2. The user first connects to the Shiny server and opens the ShinyVar web-based application. When the application opens, the user inputs the data. The user interface elements responsible for data input from the user and result visualization are integrated into the ui.R file. A function that includes the ShinyVar pipeline was saved in server.R. Both server.R and ui.R are utilized in the ShinyVar web-based application through the source function. The final variable in each ShinyVar pipeline function is returned as the output using the return function, which is placed at the end of the pipeline. All ShinyVar function scripts are stored in a directory named “R”. The complete set of R scripts for the ShinyVar functions is publicly available on GitHub https://github.com/lee99dn/ShinyVar. Results from input data are categorized into three variant groups**: **(1) universal variants (*only_VCF1_VCF2_VCF3_VCF4.csv*), (2) likely common variants (*only_VCF1_VCF2.csv, only_VCF2_VCF3.csv, only_VCF3_VCF4.csv, only_VCF1_VCF2_VCF3.csv, only_VCF2_VCF3_VCF4.csv, only_VCF1_VCF3.csv, only_VCF1_VCF3_VCF4.csv, only_VCF1_VCF4.csv, only_VCF1_VCF2_VCF4.csv, only_VCF2_VCF4.csv***)**, and (3) unique variants **(***only_VCF1.csv, only_VCF2.csv, only_VCF3.csv, only_VCF4.csv*). All results are displayed in a Venn diagram. Finally, the three variant groups and all variants (All.csv) are compressed in a file named summary.zip.

### Structure modeling

Only variants in the HA and NA genes of the four SRA samples (Subtype A-H1N1pdm09 2024, Subtype A-H3N2 2024, lineage B/Victoria 2025 and lineage B/Yamagata 2025; Table 1) were selected and annotated using SnpEff (Cingolani et al., 2012).

Protein structures were modeled with SWISS-MODEL (Waterhouse et al., 2018) For each subtype and lineage, SWISS-MODEL searched for homology templates using the reference FASTA sequence. Then, the mutant FASTA sequence was created using variants from the VCF file and the mutant structure was modeled using the wild-type template of each subtype and lineage. Protein models were assessed using QMEANDisCo Global scores greater than 0.6 as a cut-off for molecular docking (Studer et al., 2020), and Global Model Quality Estimate (GMQE) scores greater than 0.7 were considered indicative of reliable structures (Waterhouse et al., 2018).

### Molecular docking

The wild-type and mutant HA genes of the four influenza subtypes were modeled and docked with the 5J8 antibody **(**PDB ID: 4M5Y**)**. Docking was performed specifically on residues of the HA chain A of each influenza A (Hong et al., 2013) and influenza B subtype. For the 5J8 antibody, the following residues on the heavy chain were used for docking: 32, 52, 97, and 100 (Hong et al., 2013). Docking of the HA-5J8 complexes was performed using HADDOCK 2.4 (Dominguez, Boelens & Bonvin, 2003), and the top-ranked docking models were selected based on HADDOCK scores. Binding free energy (ΔG, in kcal·mol^−1^) of the selected complexes was calculated using the PRODIGY web server (Xue et al., 2016). Both wild-type and mutant NA genes of the four subtypes were docked with oseltamivir, using a set of binding site residues from chain A (Phanich et al., 2015; Khan et al., 2025). The grid box was centered at specific coordinates, and the set size was calculated using the get_position command based on the set of binding site residues for each subtype, derived from chain A of the wild-type template in PyMOL (Schrödinger LLC, 2015). NA protein structures of the four subtypes were prepared as receptors using the mk_prepare_receptor.py script, and oseltamivir was prepared as the ligand using mk_prepare_ligand.py from AutoDock Vina (Morris et al., 2009). Protein–ligand docking was performed using AutoDock Vina (Morris et al., 2009). To obtain more consistent docking results, the grid box was centered at the coordinates, and the set size and exhaustiveness parameters were set to 100 (Morris et al., 2009). The best docking complex was selected based on the lowest ΔG and RMSD values of each subtype. An increase in the ΔG of mutant docking indicated less compact binding of the protein.

## Results

### ShinyVar

**Figure 3:**
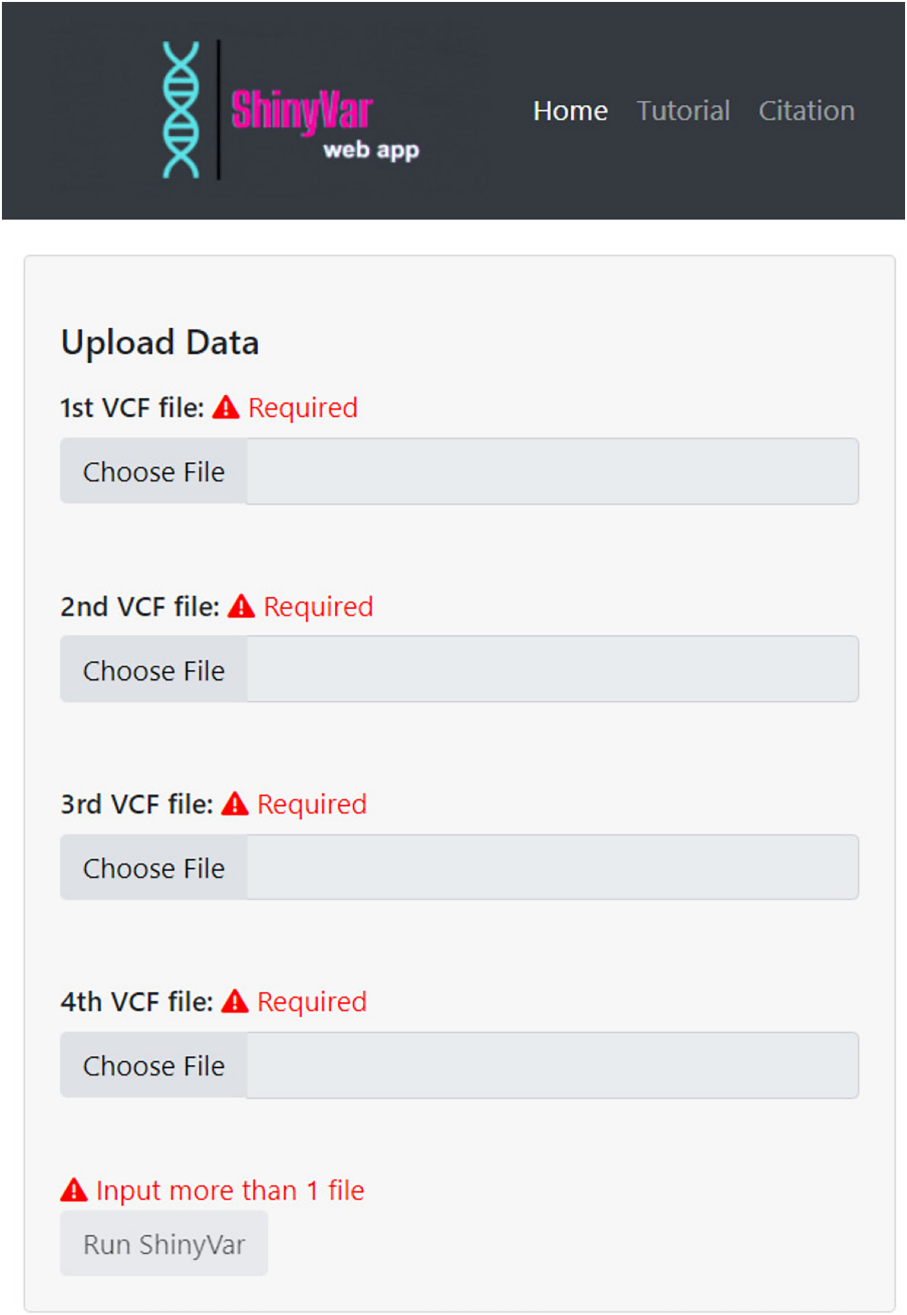
Pre-upload interface of ShinyVar.

**Figure 4:**
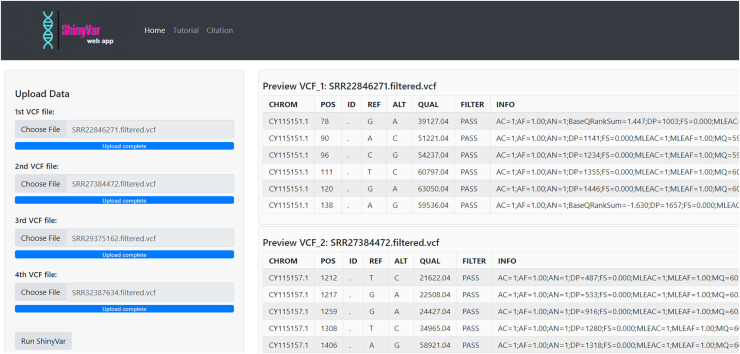
Post-upload interface of ShinyVar.

**Figure 5:**
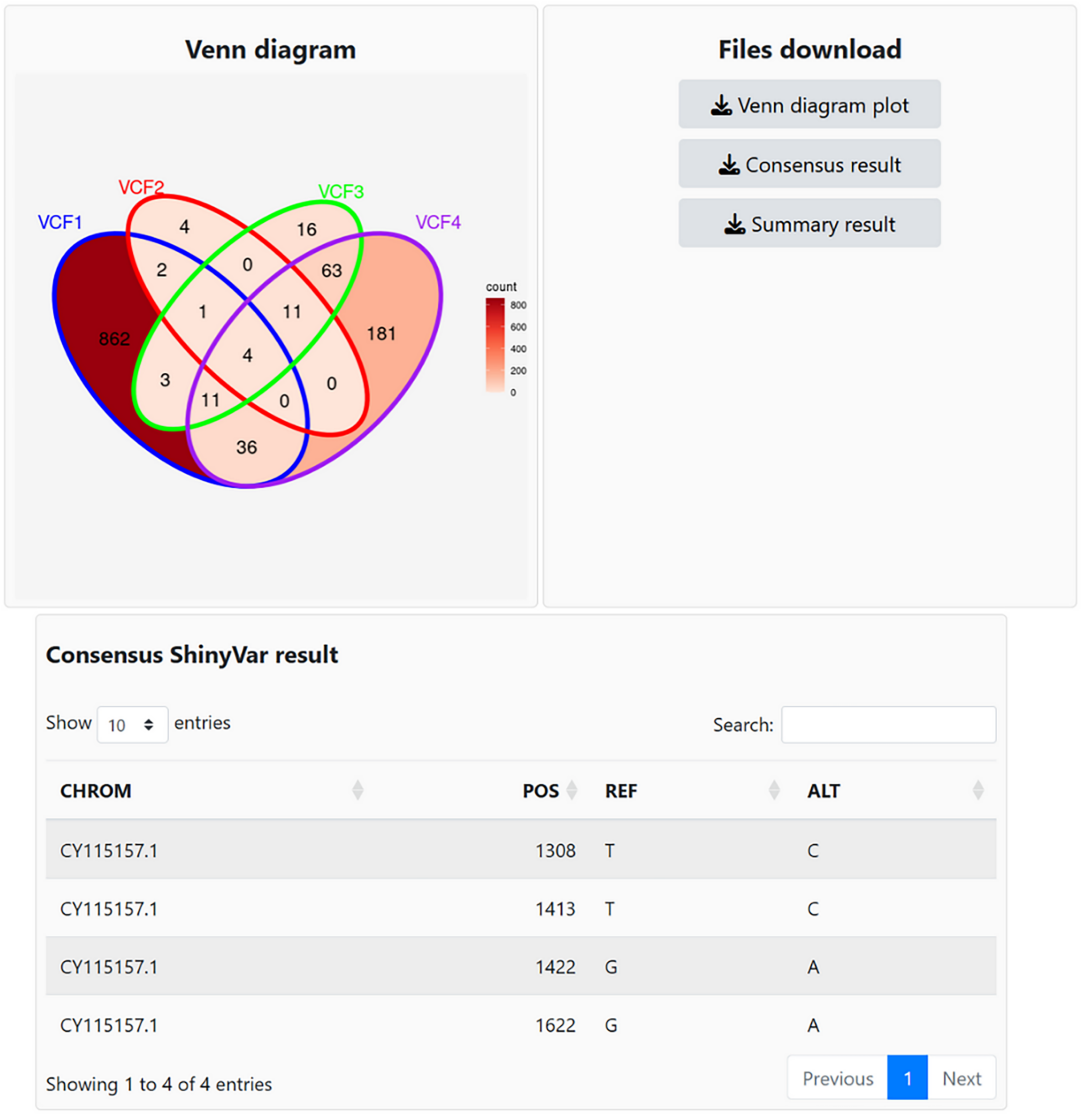
Result interface of ShinyVar.

The initial interface of ShinyVar consists of three main tabs: Home, Tutorial, and Citation. Before proceeding with any analysis, users are required to upload at least two VCF files, as illustrated in Fig. 3. Once the VCF files are uploaded, a preview of the uploaded data is displayed in a tabular format on the right side of the upload panel, allowing users to verify their input before initiating the analysis (Fig. 4) by clicking the Run ShinyVar button. The results presented in the right panel include a Venn diagram showing variant intersections, downloadable result files, and a consensus variant table generated by ShinyVar (Fig. 5).

ShinyVar supports comparative analysis of up to four samples to explore variant profiles across the complete eight genomic segments of the IFV. When the identified variants of the four viral subtypes were categorized (Figs. S1, S2, S3, and S4), no universal variants were found for the A/H1N1pdm09 subtype, while 59 likely common variants and 847 unique variants were identified. The A/H3N2 subtype showed no universal variants, 139 likely common variants and 1,043 unique variants. The B/Victoria lineage showed four universal variants, 127 likely common variants, and 1,063 unique variants. Notably, the B/Yamagata lineage exhibited a high number of universal variants (83), along with 656 likely common variants and 213 unique variants. This classification provides insight into both conserved and divergent genomic regions across IFV subpopulations over multiple years.

### Structure modeling and molecular docking

For all subtypes, only the fourth sample was selected, focusing exclusively on the HA and NA genes of the IFV. Non-synonymous variants were detected only in the fourth samples of the H1N1pdm09 and B/Victoria groups (Table 3). These variants were subsequently used for structural modeling and molecular docking analyses with the 5J8 antibody (for HA) and oseltamivir (for NA).

**Table 3:** Mutation in FASTA sequence. All mutation positions in HA and NA were reported using signal peptide–inclusive numbering, based on the full-length reference sequences (A/H1N1pdm09 subtype HA: QRV63266.1; NA: QRV63257.1; B/Victoria lineage HA: AFH57909.1; NA: AFH57913.1). This approach ensures coordinate consistency between nucleotide-level variants in the VCF files and the corresponding amino acid changes. For HA, the signal peptide comprises approximately the first 17 amino acids; therefore, residue numbering begins at the start codon (Met-1) rather than at the mature HA1 region. Users comparing results with external databases such as GISAID, FluSurver, or literature reports should note that those sources typically use signal peptide–excluded numbering. Position offsets can be adjusted accordingly to maintain consistency.

Subtype and lineage	Mutation	Reference accession number and length	Signal peptide region
HA_A/H1N1pdm09 subtype mutant	p.Lys71Gln, p.Thr137Ala, p.Lys186Gln, p.Ala203Thr, p.Glu241Ala, p.Arg276Lys, p.Lys325Arg, p.Ile435Val	MW626062.1 1,752 bp	1–17 bp
NA_A/H1N1pdm09 subtype mutant	p.Ile264Thr, p.Val453Met, p.Lys469Asn	MW626056.1 1,433 bp	0 bp
HA_B/Victoria lineage mutant	p.Ile132Val, p.Thr136Ile, p.Ala142Thr, p.Lys151Glu, p.Pro159Leu, p.Asn165Lys, p.Asp179_Asn181del, p.Gly199Glu, p.Asp212Glu, p.Lys218Arg, p.Arg294Lys, p.Ala347Val	CY115151.1 1,795 bp	1–18 bp
NA_B/Victoria lineage mutant	p.Pro42Gln, p.Ile45Thr, p.Val71Leu/Ala, p.Ser295Arg, p.Asn340Asp, p.Lys343Glu, p.Glu358Lys, p.Asp384Gly, p.Ala395Val, p.Ser397Asn, p.Val401Ile	CY115153.1 1,512 bp	0 bp

**Figure 6:**
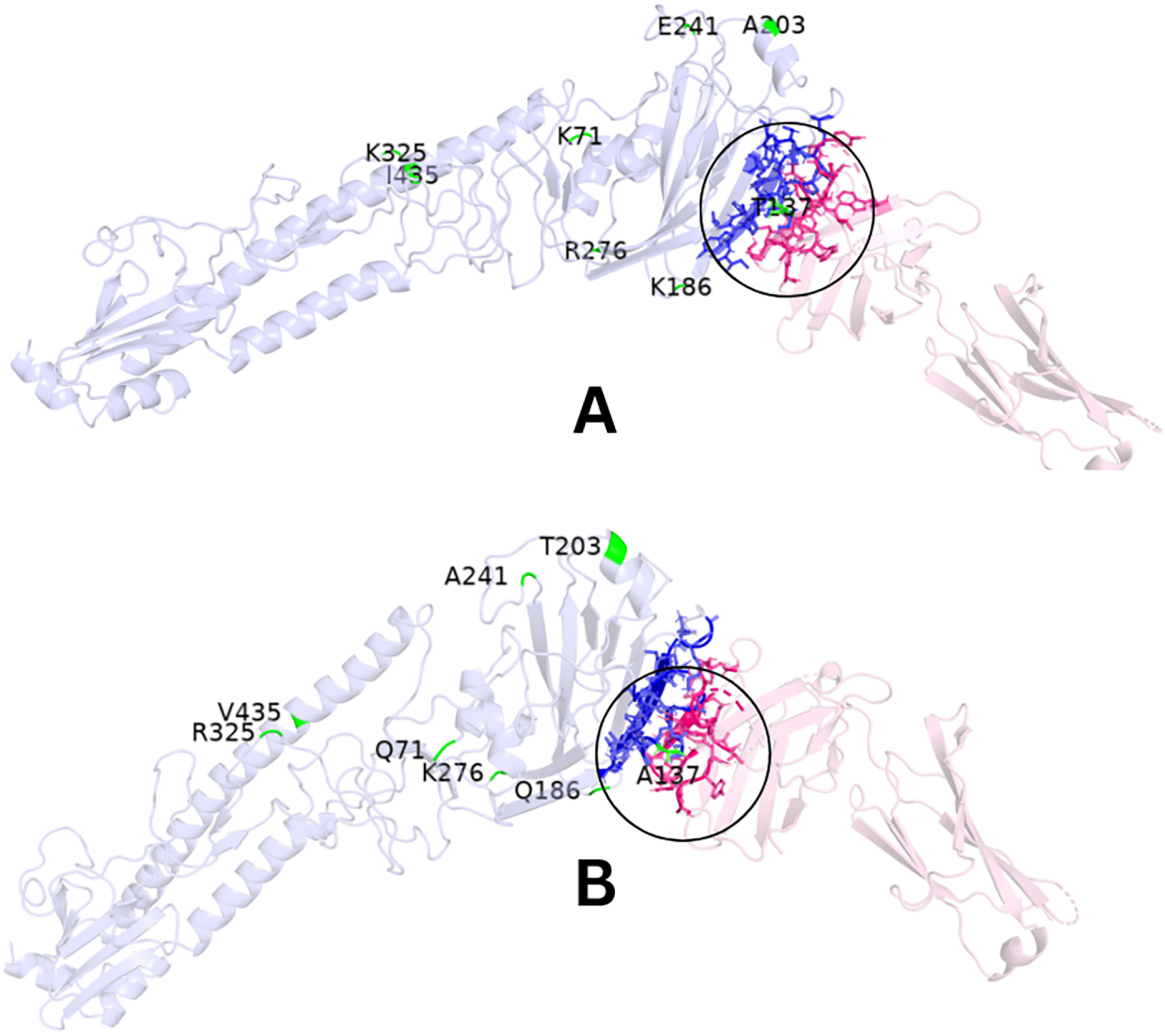
Structural representation of the wild-type and mutant HA/H1N1 pdm09-5J8 antibody complexes. The hemagglutinin (HA) protein from the H1N1 pdm09 influenza subtype is shown in gray ribbon, and the 5J8 antibody is depicted in light orange ribbon. The HA binding site is highlighted in dark blue, while the 5J8 antibody binding site is indicated in dark red. Mutation sites are marked in green ribbon. (A) Structure of the wild-type HA-5J8 antibody complex (ΔG = −11.3 kcal·mol^−1^). (B) Structure of the mutant HA-5J8 antibody complex (ΔG = −10.9 kcal·mol^−1^).

**Figure 7:**
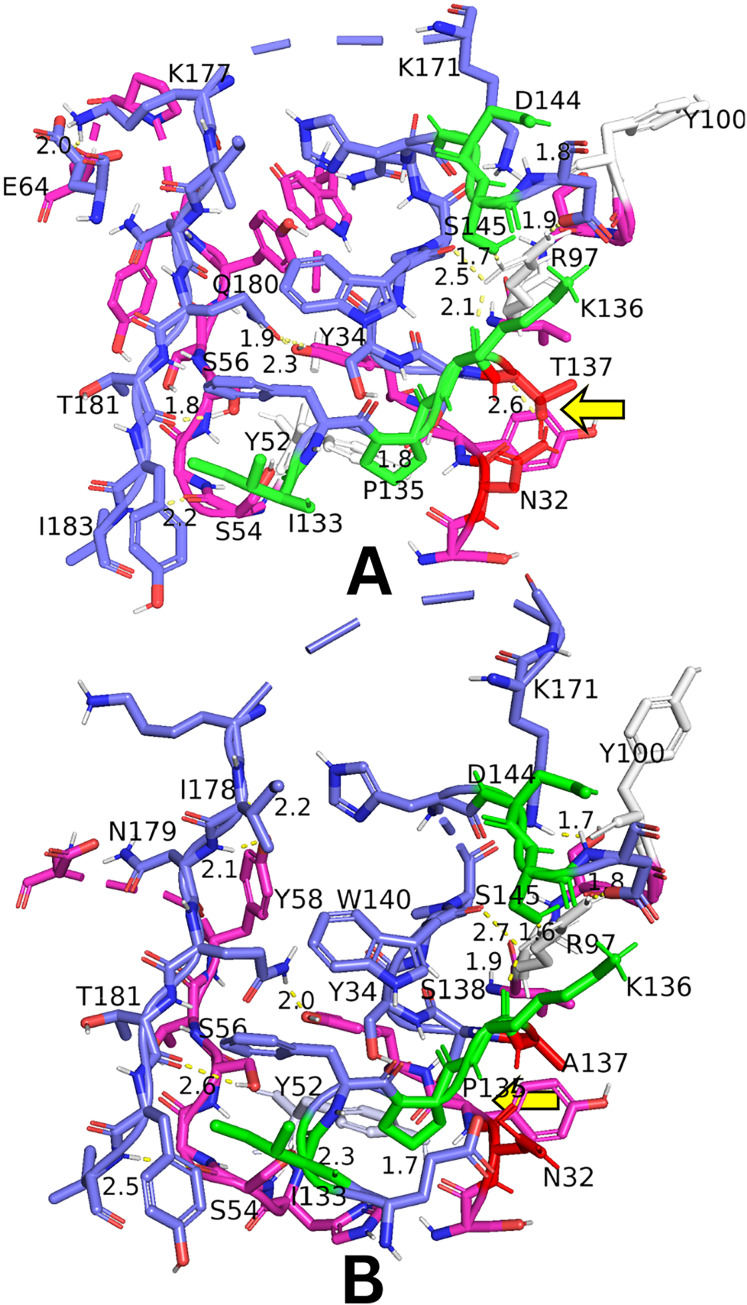
Structural representation of binding sites of the wild-type and mutant HA/H1N1pdm09-5J8 antibody complex. The hemagglutinin (HA) protein binding site from the H1N1pdm09 influenza subtype is shown with carbon (C) atoms in light purple sticks, and the 5J8 antibody with C atoms in pink sticks. Oxygen (O) atoms are depicted in dark blue, and hydrogen (H) atoms are shown in white for both structures. The key binding residues of HA are highlighted in green chains, and those of the 5J8 antibody are highlighted in white chains. Mutated residues in HA and interacting 5J8 residues are shown in red chains. Distances between interacting atoms are indicated by yellow dashed lines (measured in angstroms, Å). (A) Structure of the wild-type HA-5J8 antibody complex (ΔG = −11.3 kcal·mol^−1^). (B) Structure of the mutant HA-5J8 antibody complex (ΔG = −10.9 kcal·mol^−1^).

The protein structures of HA_A/H1N1pdm09 wild-type, HA_A/H1N1pdm09 mutant, NA_A/H1N1pdm09 wild-type, and NA_A/H1N1pdm09 mutant (Table 4) were deemed reliable for molecular docking, as all models achieved QMEANDisCo Global scores >0.6 (Studer et al., 2020) and GMQE scores >0.7 (Waterhouse et al., 2018). Structural representations of the wild-type and mutant HA/H1N1pdm09-5J8 antibody complexes are shown in Fig. 6. The wild-type and mutant HA genes of the A/H1N1pdm09 influenza subtype were modeled and docked with the 5J8 antibody. Docking was performed specifically on the following residues of the HA A chain: 133, 135, 136, 137, 143, 144, 145, 153, 189, 190, 192, 193, 194, 222, 225 and 226. The residues on the heavy chain of the 5J8 antibody used for docking included 32, 52, 97, and 100 (Hong et al., 2013). Docking of the HA-5J8 complexes was performed using HADDOCK 2.4 (Dominguez, Boelens & Bonvin, 2003), and the top-ranked docking models were selected based on HADDOCK scores. The ΔG of the selected complexes was calculated using the PRODIGY web server (Xue et al., 2016). Detailed visualizations of the binding sites for the wild-type and mutant HA/H1N1pdm09-5J8 antibody complexes are shown in Fig. 7. The ΔG of the wild-type HA-5J8 antibody complex was −11.3 kcal·mol^−1^, whereas the mutant HA-5J8 complex exhibited a slightly lower ΔG of −10.9 kcal·mol^−1^. This difference suggests that the mutation may weaken the interaction between the HA protein and the 5J8 antibody.

**Table 4:** Protein structure description.

Subtype and lineage	PDB template ID	QmEANDisCo global	GMQE
HA_A/H1N1pdm09 subtype wildtype	7kna	0.76 ± 0.05	0.74
HA_A/H1N1pdm09 subtype mutant	HA_A/H1N1pdm09 subtype wildtype	0.76 ± 0.05	0.73
NA_A/H1N1pdm09 subtype wildtype	3ti3	0.93 ± 0.05	0.80
NA_A/H1N1pdm09 subtype mutant	NA_A/H1N1pdm09 subtype wildtype	0.93 ± 0.05	0.79
HA_B/Victoria lineage wildtype	8sj9	0.52 ± 0.05	0.48
HA_B/Victoria lineage mutant	HA_B/Victoria lineage wildtype	0.51 ± 0.05	0.57
NA_B/Victoria lineage wildtype	4cpm	0.91 ± 0.05	0.87
NA_B/Victoria lineage mutant	NA_B/Victoria lineage wildtype	0.90 ± 0.05	0.78

**Figure 8:**
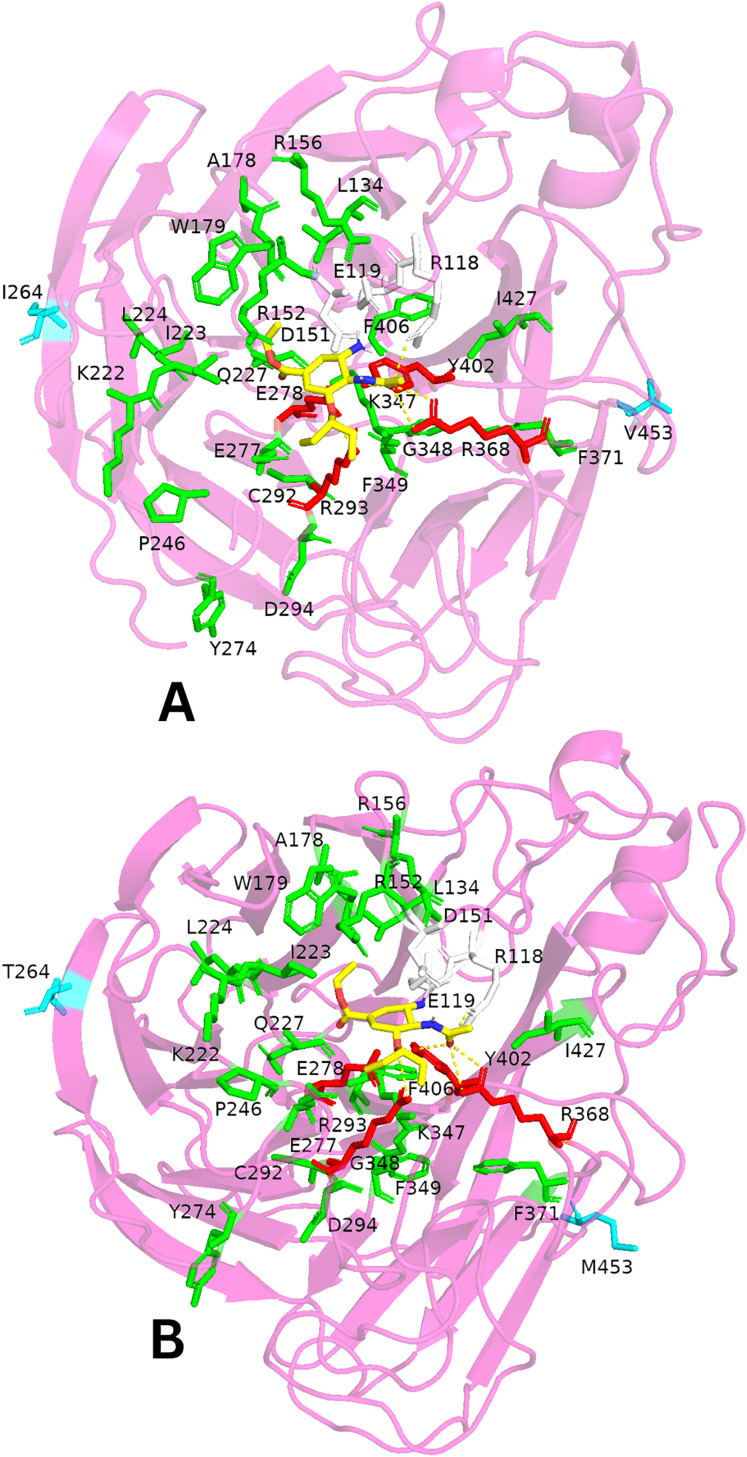
Structural representation of the wild-type and mutant NA/H1N1 pdm09—oseltamivir complexes. The neuraminidase (NA) protein from the H1N1 pdm09 influenza subtype is shown as a purple ribbon, and oseltamivir is represented as sticks with carbon (C) atoms in yellow, oxygen (O) atoms in dark blue, and hydrogen (H) atoms in white. Key binding residues of NA are highlighted in green chains. Hydrogen bonds between NA and oseltamivir are indicated by red chains, and intersections between key binding residues and hydrogen bonds are shown in white. Mutation sites within NA are marked with blue ribbons and sticks. (A) Structure of the wild-type NA-oseltamivir complex (ΔG = −6.5 kcal·mol^−1^). (B) Structure of the mutant NA-oseltamivir complex (ΔG = −6.4 kcal·mol^−1^).

**Figure 9:**
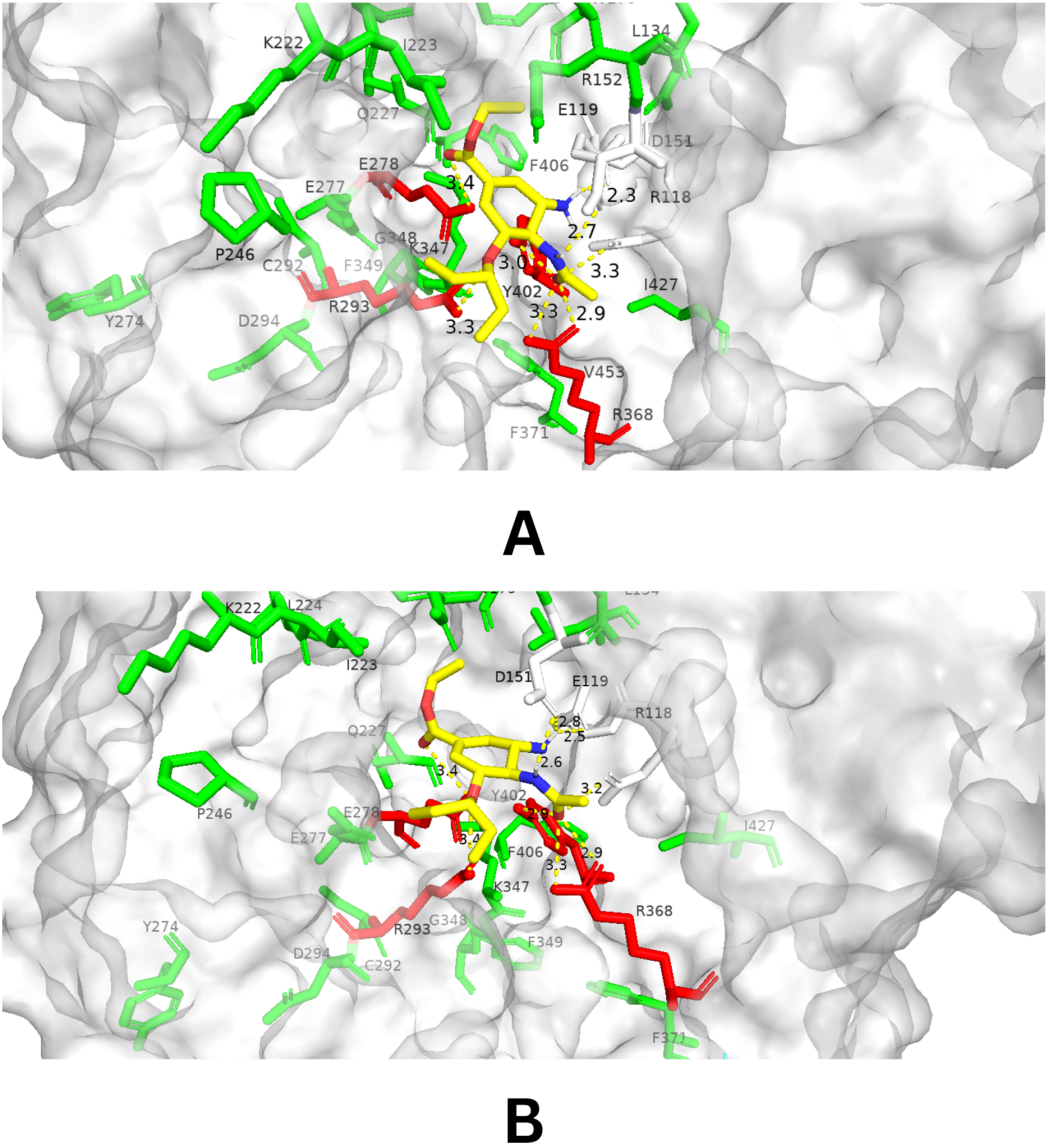
Structural representation of binding sites of the wild-type and mutant NA/H1N1pdm09—oseltamivir complexes. Oseltamivir is represented as sticks with carbon (C) atoms in yellow, oxygen (O) atoms in dark blue, and hydrogen (H) atoms in white. Key binding residues of NA are highlighted in green chains. Hydrogen bonds between NA and oseltamivir are indicated by red chains, and intersections between key binding residues and hydrogen bonds are shown in white. Mutation sites within NA are marked with blue ribbons and sticks. Distances between interacting atoms are indicated by yellow dashed lines (measured in angstroms, Å). (A) Structure of the wild-type NA-oseltamivir complex (ΔG = −6.5 kcal·mol^−1^). (B) Structure of the mutant NA-oseltamivir complex (ΔG = −6.4 kcal·mol^−1^).

Structural representations of the wild-type and mutant NA/H1N1pdm09-oseltamivir complexes are shown in Fig. 8. The NA the wild-type and mutant genes of the A/H1N1pdm09 subtype were docked with oseltamivir using the following set of binding site residues from chain A: 118, 119, 134, 151, 152, 156, 178, 179, 222, 223, 224, 227, 246, 274, 277, 292, 294, 347, 348, 349, 371, 406, and 427 (Khan et al., 2025). The grid box was centered at coordinates x = 29.812, y = 18.585, z = −22.634 and size was set to x = 20, y = 20, z = 20. The parameters were calculated using the command get_position in PyMOL with the set of binding site residues from chain A of the wild-type template (Schrödinger LLC, 2015). Protein–ligand docking of NA from the H1N1pdm09 subtype was performed using AutoDock Vina (Morris et al., 2009). Detailed vizualizations of the binding sites for both complexes are shown in Fig. 9. The ΔG of the wild-type NA-oseltamivir complex was −6.5 kcal·mol^−1^, whereas the mutant NA-oseltamivir complex exhibited a slightly lower ΔG of −6.4 kcal·mol^−1^. This difference suggests that the mutation may weaken the interaction between the NA protein and oseltamivir.

**Figure 10:**
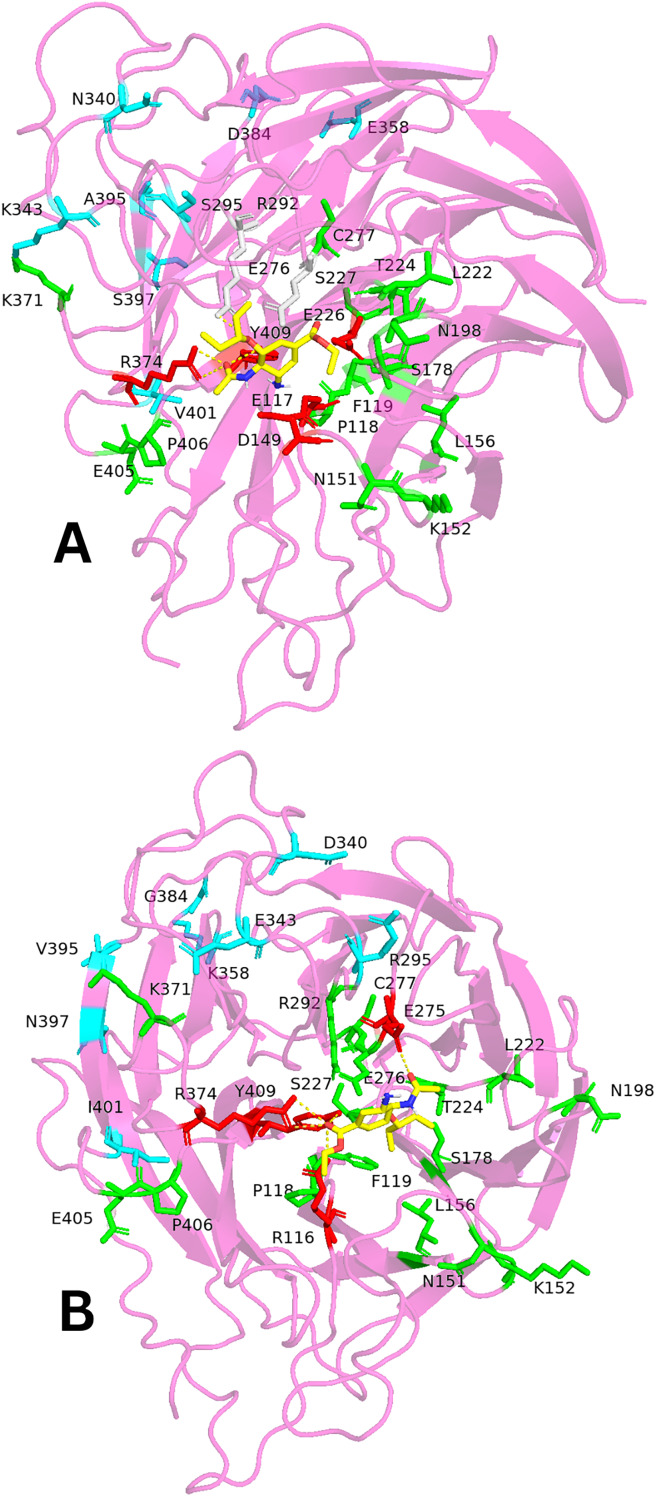
Structural representation of the wild-type and mutant NA_B/Victoria-oseltamivir complexes. The neuraminidase (NA) protein from the B/Victoria influenza subtype is shown as a purple ribbon, and oseltamivir is represented as sticks with carbon (C) atoms in yellow, oxygen (O) atoms in dark blue, and hydrogen (H) atoms in white. Key binding residues of NA are highlighted in green chains. Hydrogen bonds between NA and oseltamivir are indicated by red chains, and intersections between key binding residues and hydrogen bonds are shown in white. Mutation sites within NA are marked with blue ribbons and sticks. (A) Structure of the wild-type NA_B/Victoria-oseltamivir complex (ΔG = −6.7 kcal·mol^−1^). (B) Structure of the mutant NA_B/Victoria-oseltamivir complex (ΔG = −6.3 kcal·mol^−1^).

No variants were found in the HA and NA genes of the A/H3N2 subtype; therefore, structure modeling and molecular docking were not performed. Table 4 shows that reliable structure modeling could not be achieved for the HA_B/Victoria lineage models and they were therefore excluded from downstream analysis, as they failed to meet the GMQE threshold for model reliability and the QMEANDisCo Global cut-off for docking accuracy. However, the NA_B/Victoria lineage models met both the QMEANDisCo Global cut-off and the GMQE threshold, indicating structural reliability suitable for docking analysis. Structural representations of the wild-type and mutant NA_B/Victoria-oseltamivir complexes are presented in Fig. 10.

**Figure 11:**
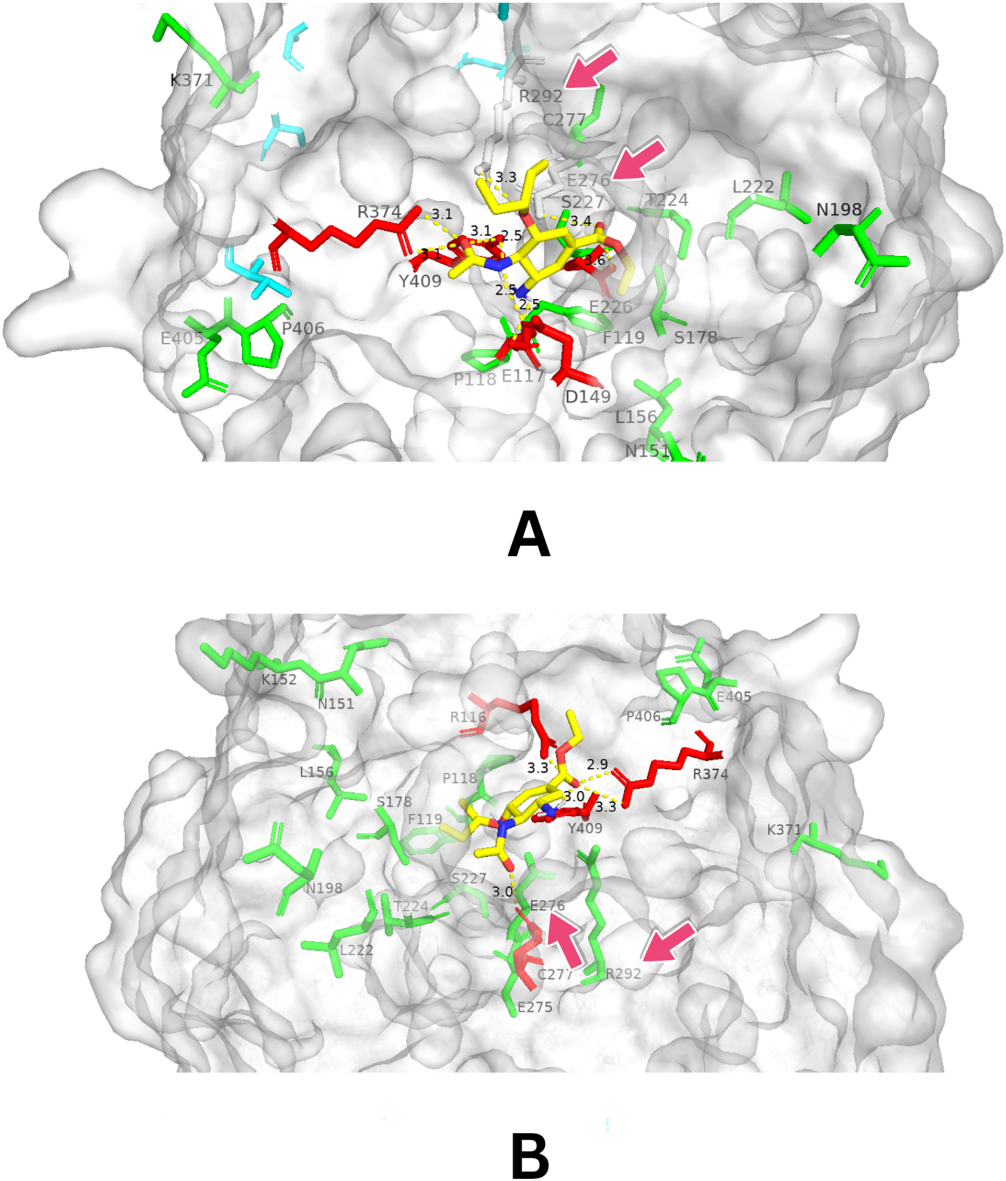
Structural representation of binding sites of the wild-type and mutant NA_B/Victoria-oseltamivir complexes. Oseltamivir is represented as sticks with carbon (C) atoms in yellow, oxygen (O) atoms in dark blue, and hydrogen (H) atoms in white. Key binding residues of NA are highlighted in green chains. Hydrogen bonds between NA and oseltamivir are indicated by red chains, and intersections between key binding residues and hydrogen bonds are shown in white. Mutation sites within NA are marked with blue ribbons and sticks. Distances between interacting atoms are indicated by yellow dashed lines (measured in angstroms, Å). (A) Structure of the wild-type NA_B/Victoria-oseltamivir complex (ΔG = −6.7 kcal·mol^−1^). (B) Structure of the mutant NA_B/Victoria-oseltamivir complex (ΔG = −6.3 kcal·mol^−1^).

The wild-type and mutant NA genes of the B/Victoria lineage were both docked with oseltamivir using NA chain A residues 118, 119, 151, 152, 156, 178, 198, 222, 224, 227, 276, 277, 292, 371, 405 and 406 (Phanich et al., 2015). Docking was performed using AutoDock Vina with the grid center set to x = 56.29, y = −69.755, z = 5.230, a box size of 20, 20, 20, and an exhaustiveness of 100. The coordinates of the docking center were determined using PyMOL (Schrödinger LLC, 2015) and were based on the modeled wild-type NA structure. The corresponding binding interfaces of both complexes are presented in Fig. 11. The ΔG of the wild-type NA-oseltamivir complex was −6.7 kcal·mol^−1^, whereas the mutant NA-oseltamivir exhibited a slightly lower ΔG of −6.3 kcal·mol^−1^. This difference suggests that the mutation may weaken the interaction between the NA protein and oseltamivir. Lastly, variants were not found in the HA and NA genes of the B/Yamagata lineage; therefore, structure modeling and molecular docking were not performed.

## Discussion

The ShinyVar web application is developed in R with a Shiny-based interface, enabling rapid and interactive comparison of IFV variant profiles. ShinyVar enables efficient variant visualization through a user-friendly web interface. In contrast, manual comparison using the same variant dataset is time consuming even in non-distracting conditions and the output may contain human errors. Although similar software packages exist, ShinyVar offers notable advantages over existing software. For instance, BEDtools (Quinlan & Hall, 2010) is limited to comparing two files per operation and relies heavily on command-line instructions. In contrast, ShinyVar allows simultaneous comparison of up to four variant files and facilitates visualization of IFV mutations by analyzing variant profiles across subpopulations through direct file uploads *via* a user-friendly web interface. In terms of runtime efficiency and accessibility, ShinyVar outperforms existing command line tools. For example, VCFtools (Danecek et al., 2011), can compare two subpopulations (two files) whereas ShinyVar can compare four subpopulations. Although VCFtools is accessible through graphical user interfaces such as the Galaxy server, variant grouping within sample cohorts remains a manual process. ShinyVar overcomes this limitation by offering the advantage of automated variant grouping (*e.g*., identifying unique variants, likely common variants and universal variants). Furthermore, ShinyVar supports the real-time visualization of variant profiles, a feature not available in VCFtools. Another tool, SNPer (Sangket et al., 2015), enables variant comparison similar to ShinyVar but is limited to comparing two files per operation, operates exclusively *via* the command line and, accepts only comma-separated value (CSV) files. In contrast, ShinyVar offers several advantages: it is a fully web-based application that requires no command-line expertise, supports the direct input of standard VCF files, and eliminates the need for additional software installation. These features enhance its accessibility and user-friendliness. Also, ShinyVar supports the analysis of up to four variant callers, enabling a comprehensive population-level variant comparison.

VCF is considered the gold standard for variant analysis because it is specifically designed to store detailed information about genetic variations. As a text-based, tab-delimited format, it can represent multiple variants across large genomic regions and is widely supported by comparative tools such as ShinyVar, BEDTools and VCFtools. Importantly, the INFO column in a VCF file can incorporate annotation data generated by software such as SnpEff, enabling the integration of functional and structural variant information. In contrast, FASTA files are intended for storing biological sequences in plain-text format and do not support multiple variations at the same genomic position. Consequently, they are not suitable for identifying or annotating variant-specific effects on amino acid sequences. Moreover, pairwise or multiple sequence alignments can alter the positional reference of nucleotides, making it difficult to maintain consistent genomic coordinates. The VCF format, by comparison, preserves strict positional consistency relative to the reference genome, thereby enabling accurate annotation and reliable variant interpretation.

ShinyVar focuses on comparative variant detection and visualization from multiple VCF files. Users may perform variant annotation either before or after analysis in ShinyVar using compatible tools such as SnpEff, which generate protein-level and functional site annotations and are widely available through command-line interfaces and graphical platforms (*e.g*., Galaxy). ShinyVar does not perform direct functional annotation because the application is hosted on a ShinyApps server that does not support the computational requirements of annotation tools such as SnpEff. In addition, biological interpretation of variants (*e.g*., antigenic site mapping, receptor-binding residues, or resistance-associated mutations) should be conducted using external tools alongside ShinyVar results.

As shown in Figs. S1, S2, S3, and S4, ShinyVar revealed that the B/Victoria lineage 2025 had 181 unique variants, the A/H1N1pdm09 subtype 2024 had 101 unique variants, the A/H3N2 subtype 2024 had 37 unique variants, and the B/Yamagata lineage 2025 had 7 unique variants. Structural analysis focused on the HA and NA proteins of the A/H1N1pdm09 subtype and the B/Victoria lineage. Eight non-synonymous mutations were identified in HA_A/H1N1pdm09, and three in NA_A/H1N1pdm09. In the B/Victoria lineage, twelve non-synonymous mutations were detected in HA, while eleven were identified in NA.

Table 3 lists the mutational residues identified in the annotated VCFs (available in Supplemental File) and used for homology modeling with SWISS-MODEL (Waterhouse et al., 2018). Due to the limited coverage of the template, not all mutations were considered (Biasini et al., 2014; Waterhouse et al., 2018). As shown in Table 4, both the wild-type and mutant HA_B/Victoria lineage structures did not fulfill the docking criteria due to QMEANDisCo global scores below 0.6 (Studer et al., 2020) and GMQE scores below 0.7 (Waterhouse et al., 2018), which indicated unreliable structural models.

The PDB templates used for homology modeling varied in sequence coverage. Although the HA and NA templates represented full-length sequences, the resulting models were partial and included only the globular domain of HA and the catalytic domain of NA. This limitation reflects the availability of experimentally resolved structures, as transmembrane and cytoplasmic regions are often absent. Consequently, the modelled structures capture the functional domains relevant for receptor binding and inhibitor docking but may not represent the complete protein.

Docking analysis revealed protein–protein interactions in the HA_2024_H1N1pdm09-5J8 antibody complex (Figs. 6 and 7). The 5J8 antibody targets the RBS of HA, a key epitope for virus neutralization by broadly neutralizing antibodies that prevent virus binding to host receptors (Julien, Lee & Wilson, 2012). Eight non-synonymous mutations were identified in the HA_H1N1pdm09 mutant. One of these mutations, p.Thr137Ala of the HA protein, located within the antibody binding site, resulted in the loss of a hydrogen bond between residue N32 of the 5J8 antibody and the HA protein (Fig. 7). This finding suggests that the substitution may reduce binding affinity (ΔG = −11.3 kcal·mol^−1^ to ΔG = −10.9 kcal·mol^−1^) and impair the neutralization efficacy of vaccines (Koel et al., 2013). Docking analysis also revealed protein–ligand interactions in the NA_2024_H1N1pdm09-oseltamivir complex (Figs. 8 and 9). Oseltamivir is an antiviral drug that inhibits the active site of NA, which is critical for the release of viral progeny (Davies, 2010). Two mutations were identified outside the binding site and the integrity of the binding site was preserved (Dejnirattisai et al., 2021). However, the protein structure of NA indicated minimal conformational changes. Consequently, a slight decrease in binding affinity was observed (ΔG = −6.5 kcal·mol^−1^ to ΔG = −6.4 kcal·mol^−1^), which may correspond to a minor reduction in the efficacy of oseltamivir.

In contrast, docking results for the NA_2024_B/Victoria-oseltamivir complex (Figs. 10 and 11) revealed eight non-synonymous mutations, including the loss of interaction residues E276 and R292, which are key components of hydrogen bonding with oseltamivir (Khan et al., 2025). Although the mutations occurred outside the active site, structural changes were observed in the mutant complex. These results suggest that mutations far from the binding interface can still affect protein conformation and dynamics, thereby affecting binding site geometry and protein-ligand interactions (Bloom, Gong & Baltimore, 2010). Consequently, this mutant complex exhibited a greater decrease in binding affinity (ΔG = −6.7 kcal·mol^−1^ to ΔG = −6.3 kcal·mol^−1^) compared to the NA_2024_H1N1pdm09-oseltamivir models analyzed.

While ShinyVar reports variant positions using signal peptide inclusive coordinates, this may introduce minor discrepancies when comparing results with public influenza databases that use mature HA numbering. This difference does not affect the identification or interpretation of mutations, but should be considered when cross-referencing antigenic, receptor-binding, or glycosylation sites reported elsewhere. Comparison with published studies and public databases (excluding GISAID, as the GISAID EpiFlu database is currently inactive) indicated that most mutations detected by ShinyVar have been previously reported (Kolosova et al., 2022; Singh et al., 2024; Vlaicu et al., 2024; European Centre for Disease Prevention and Control, 2024; Separovic et al., 2025; Hungnes et al., 2025; Neher, 2025a, 2025b, 2025c) in circulating strains, supporting the reliability of the tool. A small proportion of variants may represent rare or potentially novel mutations (Table S3).

## Conclusions

**Figure 12:**
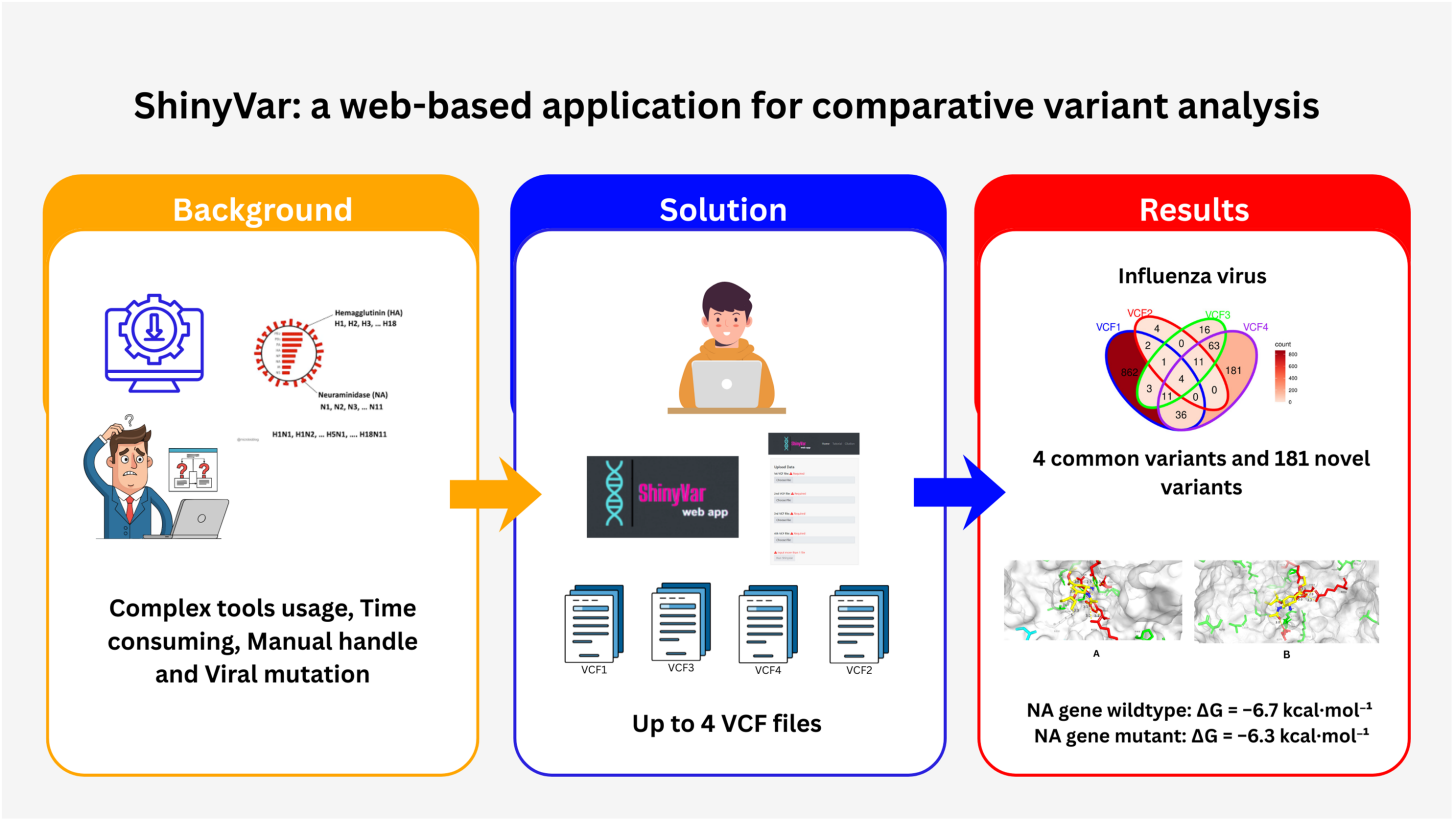
ShinyVar outline. Image Credit: Canva Pro https://www.canva.com/policies/content-license-agreement/.

ShinyVar is a web-based application that provides fast and efficient variant detection and visualization, making it suitable for monitoring IFV evolution and supporting vaccine and antiviral drug design (Fig. 12). The application automatically identifies and visualizes mutations in IFVs by comparing variant profiles across up to four subpopulations, without requiring local computational infrastructure or programming expertise. ShinyVar enables the identification of novel, common and unique variants associated with important traits such as drug resistance and virulence under selective pressure, and thus has the potential to improve our understanding of pathogen evolution, development of more effective vaccines and drugs. Its flexibility enables the analysis of quantitative variant profiles not only between IFV subpopulations, but also across other pathogens, such as Severe Acute Respiratory Syndrome Coronavirus 2 (SARS-CoV-2).

Ultimately, this tool will support a faster and more informed response to emerging infectious diseases.

## Supplemental Information

10.7717/peerj.21158/supp-1 Supplemental Information 1 Detailed information on the identified variants, including their types and annotations.

10.7717/peerj.21158/supp-2 Supplemental Information 2 An illustrative example of SRR30635452 VCF.

10.7717/peerj.21158/supp-3 Supplemental Information 3 Sorted merged dataset.

10.7717/peerj.21158/supp-4 Supplemental Information 4 Status of the mutation. Note: For the HA B/Victoria lineage, three additional positions should be included for comparison because there are different accession numbers but only two amino acid differences.

10.7717/peerj.21158/supp-5 Supplemental Information 5 Venn diagram of subtype 1 (A/H1N1pdm09). Color-coded sets indicate variant counts from VCF1 (dark blue), VCF2 (orange), VCF3 (green), and VCF4 (purple). Overlapping areas correspond to shared variants between the respective VCF files.

10.7717/peerj.21158/supp-6 Supplemental Information 6 Venn diagram of subtype 2 (A/H3N2). Color-coded sets indicate variant counts from VCF1 (dark blue), VCF2 (orange), VCF3 (green), and VCF4 (purple). Overlapping areas correspond to shared variants between the respective VCF files.

10.7717/peerj.21158/supp-7 Supplemental Information 7 Venn diagram of subtype 3 (B/Victoria). Color-coded sets indicate variant counts from VCF1 (dark blue), VCF2 (orange), VCF3 (green), and VCF4 (purple). Overlapping areas correspond to shared variants between the respective VCF files.

10.7717/peerj.21158/supp-8 Supplemental Information 8 Venn diagram of subtype 4 (B/Yamagata). Color-coded sets indicate variant counts from VCF1 (dark blue), VCF2 (orange), VCF3 (green), and VCF4 (purple). Overlapping areas correspond to shared variants between the respective VCF files.

## Additional Information and Declarations

### Data Availability

The following information was supplied regarding data availability:

The ShinyVar web app is available at shinyapps.io:

https://snperapp.shinyapps.io/ShinyVar/.

The code is available at GitHub and Zenodo:

- https://github.com/lee99dn/ShinyVar

- Danusorn Lee, & Unitsa, S. (2026). lee99dn/ShinyVar: V.1.0.0 (V.1.0.0). Zenodo. https://doi.org/10.5281/zenodo.18980297
