# Supplementary material for: ShinyVar: a web-based application for comparative Influenza variant analysis supporting structure-guided approaches to vaccine and antiviral drug design
Source: PeerJ. 2026 Jun 8;14:e21158. doi: 10.7717/peerj.21158 (PMC13256122; doi:10.7717/peerj.21158)
Supplement: Supplemental Information 2 [file peerj-14-21158-s002.docx]

**Table S1. An illustrative example of SRR30635452 VCF**

| CHROM | POS | ID | REF | ALT | QUAL | FILTER | INFO | FORMAT | SRR30635452 |
| --- | --- | --- | --- | --- | --- | --- | --- | --- | --- |
| CY121797.1 | 30 | . | TACG | T | 1309.01 | PASS | AC=1;AF=1.00;AN=1;BaseQRankSum=2.561;DP=250;FS=0.000;MLEAC=1;MLEAF=1.00;MQ=59.06;MQRankSum=0.691;QD=22.57;ReadPosRankSum=-0.900;SOR=0.744 | GT:AD:DP:GQ:PL | 1:2,450:452:99:15854,0 |
| CY121797.1 | 80 | . | A | G | 2309.01 | PASS | AC=1;AF=1.00;AN=1;BaseQRankSum=2.561;DP=250;FS=0.000;MLEAC=1;MLEAF=1.00;MQ=59.06;MQRankSum=0.691;QD=22.57;ReadPosRankSum=-0.900;SOR=0.744 | GT:AD:DP:GQ:PL | 1:2,800:452:99:  15854,0 |
| CY121799.1 | 1510 | . | A | G | 2309.01 | PASS | AC=1;AF=1.00;AN=1;BaseQRankSum=2.561;DP=250;FS=0.000;MLEAC=1;MLEAF=1.00;MQ=59.06;MQRankSum=0.691;QD=22.57;ReadPosRankSum=-0.900;SOR=0.744 | GT:AD:DP:GQ:PL | 1:2,250:452:99:  20854,0 |
