# Supplementary material for: ShinyVar: a web-based application for comparative Influenza variant analysis supporting structure-guided approaches to vaccine and antiviral drug design
Source: PeerJ. 2026 Jun 8;14:e21158. doi: 10.7717/peerj.21158 (PMC13256122; doi:10.7717/peerj.21158)
Supplement: Supplemental Information 3 [file peerj-14-21158-s003.docx]

**Table S2. Sorted merged dataset**

| CHROM | POS | REF | ALT | ID,  QUAL,  FILTER,  INFO,  FORMAT | SRR30635452 | ID,  QUAL,  FILTER,  INFO,  FORMAT | SRR26854354 | ID,  QUAL,  FILTER,  INFO,  FORMAT | SRR21931671 | ID,  QUAL,  FILTER,  INFO,  FORMAT | SRR14341061 |
| --- | --- | --- | --- | --- | --- | --- | --- | --- | --- | --- | --- |
| CY121797.1 | 30 | TACG | T | .,1309.01  , PASS ,… | 1:2,450:452:99:  15854,0 |  |  |  |  |  |  |
| CY121797.1 | 80 | A | G | .,2309.01  , PASS ,… | 1:2,800:452:99:  15854,0 | .,3309.01  , PASS ,… | 1:2,250:452:99:  15854,0 |  |  |  |  |
| CY121798.1 | 800 | T | G |  |  | .,4309.01  , PASS ,… | 1:2,250:452:99:  15854,0 | .,2312.01  , PASS ,… | 1:2,250:452:99:  15854,0 |  |  |
| CY121799.1 | 1287 | T | G |  |  | .,1309.01  , PASS ,… | 1:2,250:452:99:  15854,0 | .,3312.01  , PASS ,… | 1:2,250:452:99:  15854,0 | .,4312.01  , PASS ,… | 1:2,250:452:99:  30854,0 |
| CY121799.1 | 1510 | A | G | .,2309.01  , PASS ,… | 1:2,250:452:99:  20854,0 | .,1309.01  , PASS ,… | 1:2,250:452:99:  15854,0 | .,3312.01  , PASS ,… | 1:2,250:452:99:  15854,0 | .,4312.01  , PASS ,… | 1:2,250:452:99:  30854,0 |
