# Supplementary material for: ShinyVar: a web-based application for comparative Influenza variant analysis supporting structure-guided approaches to vaccine and antiviral drug design
Source: PeerJ. 2026 Jun 8;14:e21158. doi: 10.7717/peerj.21158 (PMC13256122; doi:10.7717/peerj.21158)
Supplement: Supplemental Information 4 — Note: For the HA B/Victoria lineage, three additional positions should be included for comparison because there are different accession numbers but only two amino acid differences. [file peerj-14-21158-s004.docx]

**Table S3. Status of the mutation.**

| **Subtype and lineage** | **Gene** | **Mutation** | **Mature Position** | **Status** |
| --- | --- | --- | --- | --- |
| A/H1N1pdm09 subtype | HA | p.Lys71Gln | **p.Lys54Gln** | Previously reported (Kolosova et al., 2022) |
|  |  | p.Thr137Ala | **p.Thr120Ala** | Previously reported (European Centre for Disease Prevention and Control, 2024) |
|  |  | p.Lys186Gln | **p.Lys169Gln** | Previously reported (Separovic et al., 2025) |
|  |  | p.Ala203Thr | **p.Ala186Thr** | Previously reported (Vlaicu et al., 2024) |
|  |  | p.Glu241Ala | **p.Glu224Ala** | Previously reported (Singh et al., 2024) |
|  |  | p.Arg276Lys | **p.Arg259Lys** | Previously reported (Singh et al., 2024) |
|  |  | p.Lys325Arg | **p.Lys308Arg** | Previously reported (Singh et al., 2024) |
|  |  | p.Ile435Val | **p.Ile418Val** | Previously reported (Brcko et al., 2024) |
|  | NA | p.Ile264Thr | **p.Ile264Thr** | Previously reported (Hungnes et al., 2025) |
|  |  | p.Val453Met | **p.Val453Met** | Previously reported (Richard Neher, 2025a) |
|  |  | p.Lys469Asn | **p.Lys469Asn** | Previously reported (Richard Neher, 2025a) |
| B/Victoria lineage | HA | p.Ile132Val | **p.Ile114Val** | Requires GISAID verification |
|  |  | p.Thr136Ile | **p.Thr118Ile** | Requires GISAID verification |
|  |  | p.Ala142Thr | **p.Ala124Thr** | Previously reported (Richard Neher, 2025b) |
|  |  | p.Lys151Glu | **p.Lys133Glu** | Previously reported (Richard Neher, 2025b) |
|  |  | p.Pro159Leu | **p.Pro141Leu** | Previously reported (Richard Neher, 2025b) |
|  |  | p.Asn165Lys | **p.Asn147Lys** | Previously reported (Richard Neher, 2025b) |
|  |  | p.Asp179_Asn181del | **p.Asp161_Asn163del** | Previously reported (Shu et al., 2020) |
|  |  | p.Gly199Glu | **p.Gly181Glu** | Previously reported (Richard Neher, 2025b) |
|  |  | p.Asp212Glu | **p.Asp194Glu** | Previously reported (Shu et al., 2020) |
|  |  | p.Lys218Arg | **p.Lys200Arg** | Previously reported (Richard Neher, 2025b) |
|  |  | p.Arg294Lys | **p.Arg276Lys** | Previously reported (Richard Neher, 2025b) |
|  |  | p.Ala347Val | **p.Ala329Val** | Requires GISAID verification |
|  | NA | p.Pro42Gln | **p.Pro42Gln** | Previously reported (Richard Neher, 2025c) |
|  |  | p.Ile45Thr | **p.Ile45Thr** | Previously reported (Richard Neher, 2025c) |
|  |  | p.Val71Leu/Ala | **p.Val71Leu/Ala** | Previously reported (Richard Neher, 2025c) |
|  |  | p.Ser295Arg | **p.Ser295Arg** | Previously reported (Richard Neher, 2025c) |
|  |  | p.Asn340Asp | **p.Asn340Asp** | Requires GISAID verification |
|  |  | p.Lys343Glu | **p.Lys343Glu** | Requires GISAID verification |
|  |  | p.Glu358Lys | **p.Glu358Lys** | Requires GISAID verification |
|  |  | p.Asp384Gly | **p.Asp384Gly** | Requires GISAID verification |
|  |  | p.Ala395Val | **p.Ala395Val** | Previously reported (Richard Neher, 2025c) |
|  |  | p.Ser397Asn | **p.Ser397Asn** | Requires GISAID verification |
|  |  | p.Val401Ile | **p.Val401Ile** | Requires GISAID verification |

Note: For the HA B/Victoria lineage, three additional positions should be included for comparison because there are different accession numbers but only two amino acid differences.
