# Supplementary figures and images for: ShinyVar: a web-based application for comparative Influenza variant analysis supporting structure-guided approaches to vaccine and antiviral drug design
Source: PeerJ. 2026 Jun 8;14:e21158. doi: 10.7717/peerj.21158 (PMC13256122; doi:10.7717/peerj.21158)

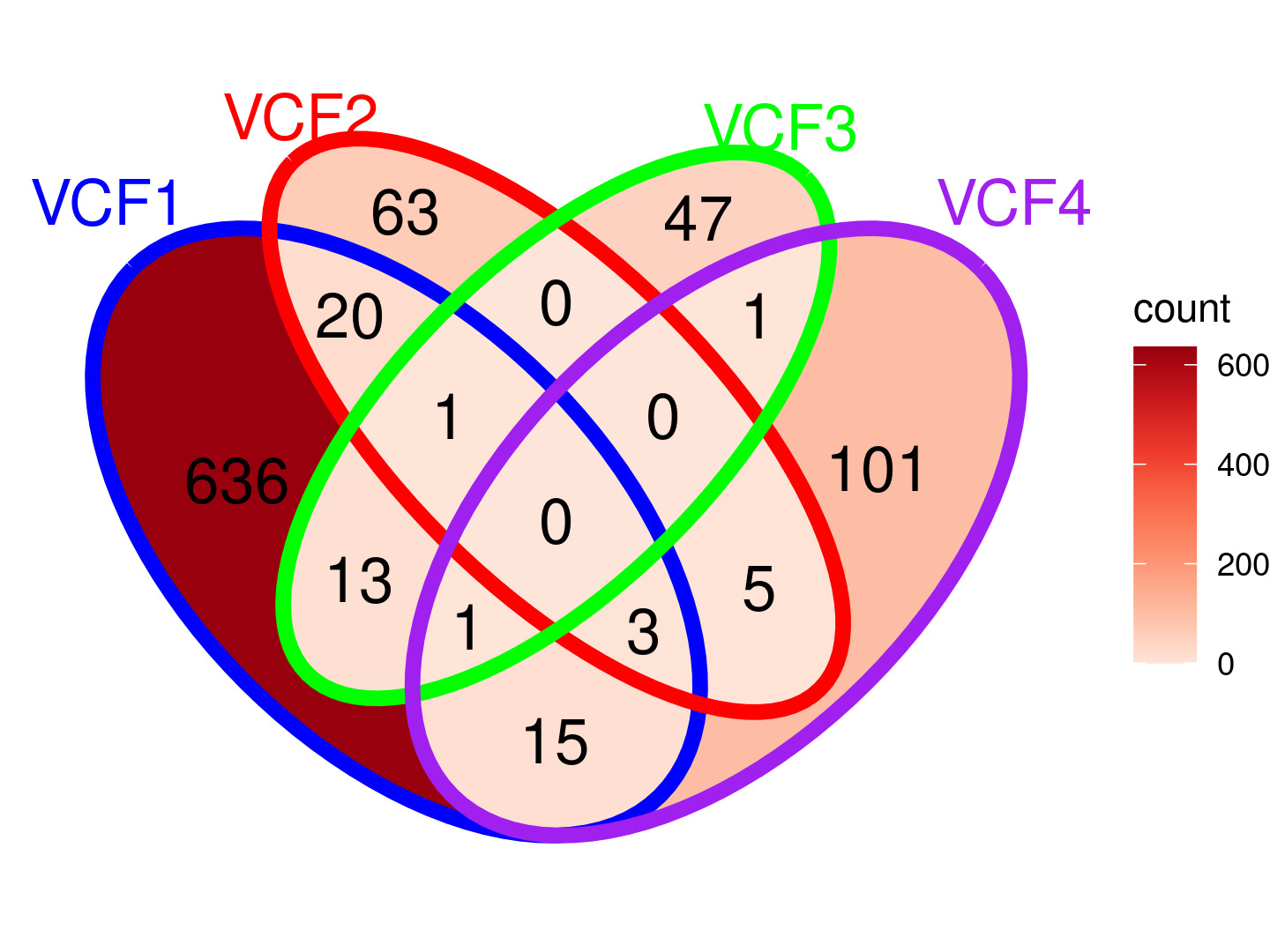

Supplement: Supplemental Information 5 — Color-coded sets indicate variant counts from VCF1 (dark blue), VCF2 (orange), VCF3 (green), and VCF4 (purple). Overlapping areas correspond to shared variants between the respective VCF files. [file peerj-14-21158-s005.jpg]

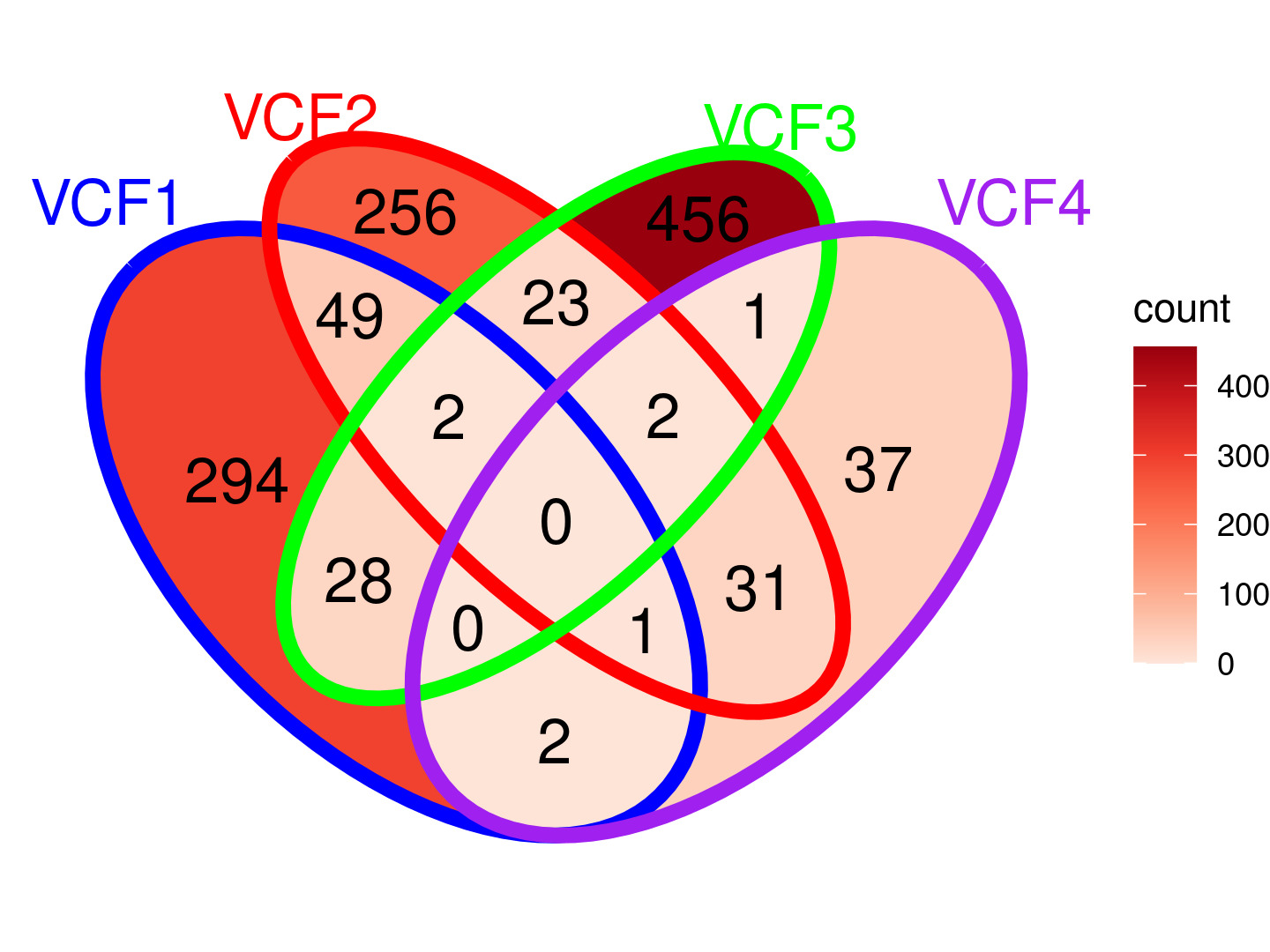

Supplement: Supplemental Information 6 — Color-coded sets indicate variant counts from VCF1 (dark blue), VCF2 (orange), VCF3 (green), and VCF4 (purple). Overlapping areas correspond to shared variants between the respective VCF files. [file peerj-14-21158-s006.jpg]

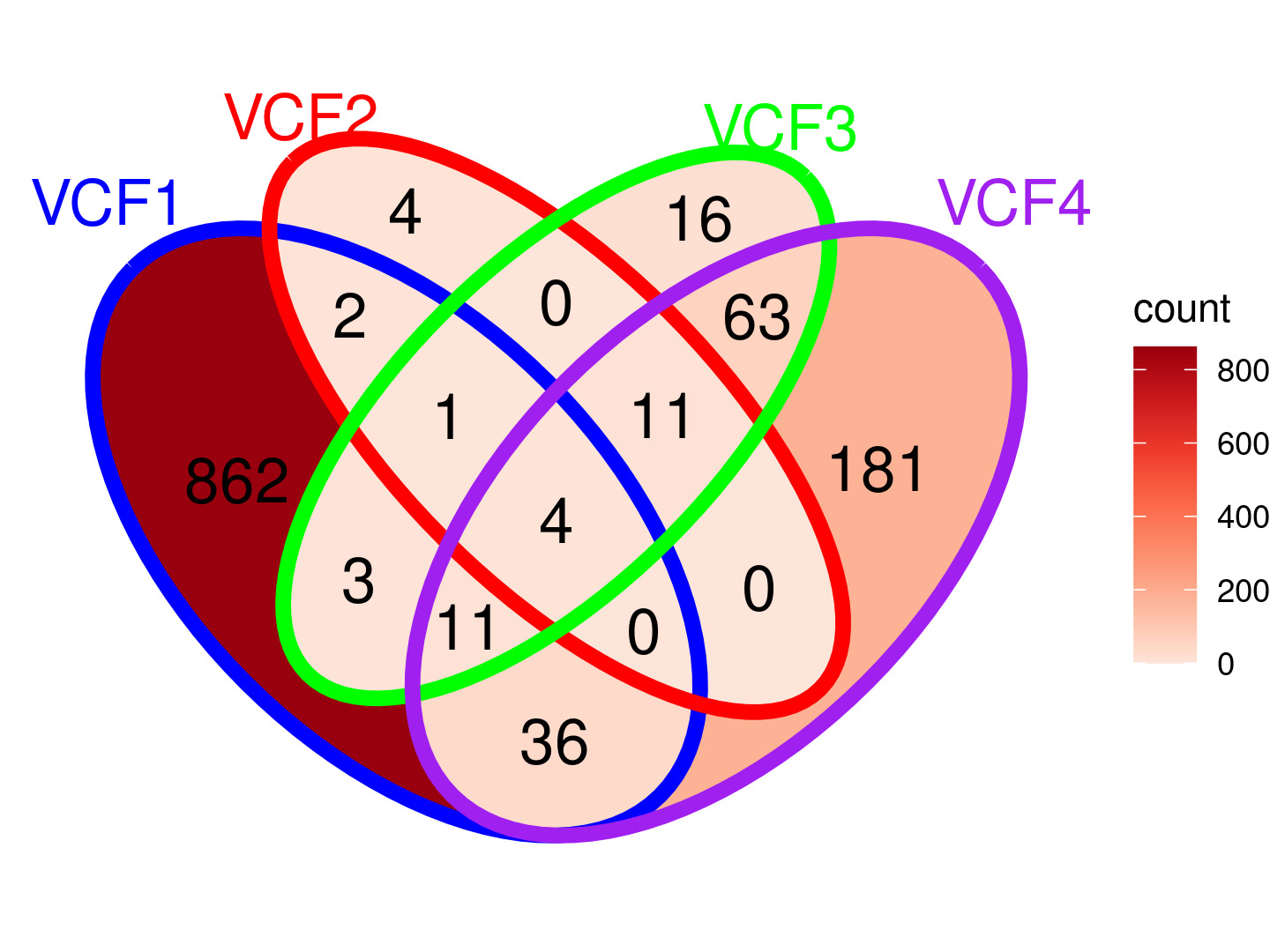

Supplement: Supplemental Information 7 — Color-coded sets indicate variant counts from VCF1 (dark blue), VCF2 (orange), VCF3 (green), and VCF4 (purple). Overlapping areas correspond to shared variants between the respective VCF files. [file peerj-14-21158-s007.jpg]

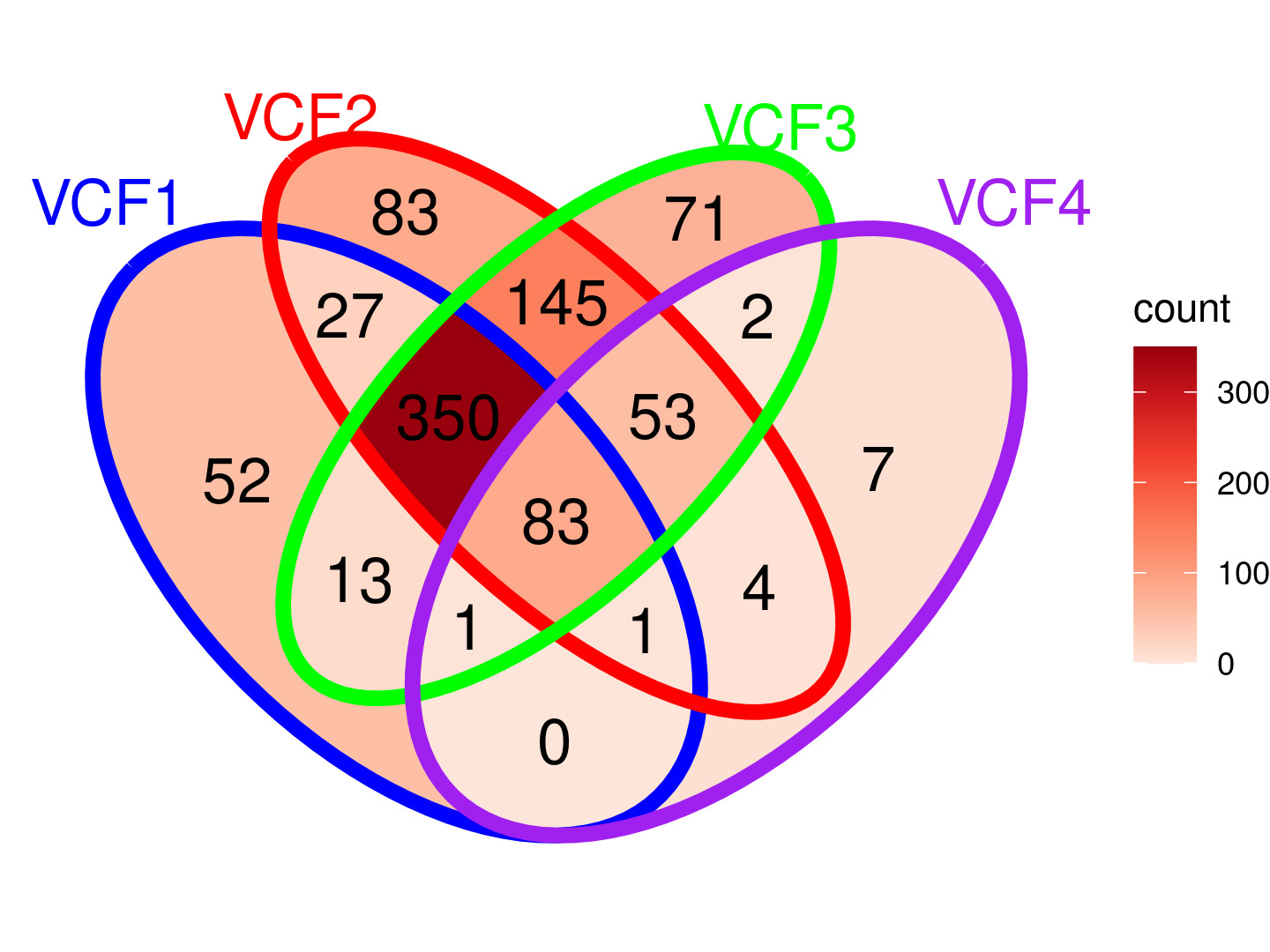

Supplement: Supplemental Information 8 — Color-coded sets indicate variant counts from VCF1 (dark blue), VCF2 (orange), VCF3 (green), and VCF4 (purple). Overlapping areas correspond to shared variants between the respective VCF files. [file peerj-14-21158-s008.jpg]
